# Intermittent Fasting and Androgen Receptor Signaling in Prostate Cancer: Metabolic Crosstalk and Therapeutic Implications

**DOI:** 10.3390/ijms27062652

**Published:** 2026-03-13

**Authors:** Grażyna Gromadzka, Maria Bendykowska

**Affiliations:** 1 Department of Biomedical Sciences, Faculty of Medicine, Collegium Medicum, Cardinal Stefan Wyszynski University in Warsaw, Wóycickiego Street 1/3, 01-938 Warsaw, Poland; 2 Military Institute of Medicine, Szaserów 128, 04-141 Warsaw, Poland

**Keywords:** prostate cancer, androgen receptor, intermittent fasting, fasting mimicking diet, diet, metabolism, AR-V7, time-restricted eating, therapy resistance, AMPK, lipogenesis

## Abstract

Prostate cancer (PCa) progression is critically driven by androgen receptor (AR) signaling, which integrates hormonal cues with metabolic programs supporting tumor growth, survival, and therapy resistance. Emerging evidence suggests that intermittent fasting (IF) and related dietary interventions—such as time-restricted eating (TRE), alternate-day fasting (ADF), and fasting-mimicking diet (FMD)—modulate systemic metabolism, including reductions in insulin and insulin-like growth factor 1 (IGF-1), and induce intracellular nutrient stress that can influence AR activity, splice variant expression (e.g., AR-V7), and downstream metabolic pathways. This systematic literature review (Scopus, PubMed, Web of Science; publications up to December 2025; search terms: “prostate cancer,” “androgen receptor,” “AR splice variants,” “intermittent fasting,” “fasting mimicking diet”, “metabolism,” “therapy resistance”) summarizes preclinical and clinical studies addressing the impact of IF on AR signaling, lipogenesis, mitochondrial function, redox homeostasis, and therapy response. Preclinical studies indicate that IF can reduce AR expression, impair nuclear translocation, modulate AR splice variants such as AR-V7 via nutrient-sensitive splicing mechanisms, and enhance sensitivity to androgen deprivation therapy and AR-targeted agents. Mechanistically, IF-induced metabolic stress engages AMP-activated protein kinase (AMPK), mechanistic target of rapamycin (mTOR), and sirtuin pathways, alters lipid and mitochondrial metabolism, and transiently increases reactive oxygen species (ROS), creating vulnerabilities in prostate tumor cells. Translational evidence suggests potential benefits of integrating IF with standard therapy, but effects may depend on fasting regimen, caloric intake, macronutrient composition, and patient metabolic context, including risk of lean mass loss. This review highlights the metabolic crosstalk between IF and AR signaling and emphasizes the need for future clinical studies incorporating biomarker-guided approaches and body composition monitoring to fully exploit this intersection for improved therapeutic outcomes in prostate cancer.

## 1. Introduction

Prostate cancer (PCa) remains one of the most prevalent malignancies in men worldwide and represents a significant cause of cancer-related mortality [1,2]. The androgen receptor (AR) is the principal driver of prostate tumorigenesis and progression, orchestrating transcriptional programs that promote proliferation, survival, and metabolic adaptation [3,4,5,6]. Therapeutic strategies targeting AR signaling, including androgen deprivation therapy (ADT) and second-generation AR pathway inhibitors such as enzalutamide, confer clinical benefit but are ultimately limited by the emergence of resistance [7,8,9].

Emerging evidence indicates that metabolic pathways are intricately linked with AR activity, and that PCa cells possess unique metabolic dependencies that support growth and survival under hormone-targeted therapy. For example, AR signaling has been shown to directly reprogram glucose, lipid, and mitochondrial metabolism and to act as a master regulator of cellular energy pathways in PCa cells [10,11,12,13].

Intermittent fasting (IF) and fasting mimicking diet (FMD) have drawn attention as dietary interventions that induce systemic metabolic stress, leading to reductions in circulating growth factors such as insulin and insulin-like growth factor 1 (IGF-1) and reprogramming intracellular nutrient sensing pathways [14,15,16,17]. Preclinical studies have shown that caloric restriction through alternate day fasting (ADF) significantly reduces AR expression and signaling in PCa models and enhances the antitumor activity of enzalutamide by impairing AR mRNA translation under amino acid limited conditions [18]. These findings suggest that IF may serve as a metabolic adjuvant to AR-targeted therapy by perturbing AR signaling and associated metabolic networks.

Despite promising preclinical data, the mechanisms by which IF influences AR signaling, metabolic adaptation, and resistance remain incompletely understood. In particular, how systemic metabolic cues intersect with intratumoral AR activity and therapeutic response represents a critical gap in translational oncology. This review synthesizes current knowledge on IF and AR signaling in PCa, focusing on AR as a metabolic sensor, the effects of IF on insulin/IGF-1 signaling and tumor metabolic reprogramming, and the therapeutic implications of targeting this metabolic crosstalk. The synthesis draws on studies conducted in PCa, findings from other malignancies, and mechanistic research on the metabolic effects of intermittent fasting independent of oncology, to delineate metabolic crosstalk and evaluate the translational therapeutic potential of targeting this axis.

## 2. Materials and Methods

A comprehensive literature review was performed to evaluate the current evidence on IF, AR signaling, and PCa metabolism. The search was conducted in Scopus, PubMed, and Web of Science databases for articles published up to December 2025.

The following search terms were used in combination: “prostate cancer”, “androgen receptor”, “AR splice variants”, “intermittent fasting”, “fasting mimicking diet”, “metabolism”, “therapy resistance”, “lipogenesis”, “mitochondria”, “redox”.

Inclusion criteria: Original research or reviews reporting mechanistic or translational studies on AR signaling and metabolic regulation in PCa; preclinical studies (cell lines, patient-derived xenografts, genetically engineered mouse models); clinical studies investigating IF, caloric restriction, or fasting-mimicking diets in men with PCa.

Exclusion criteria: Non-English articles; Studies not directly related to AR signaling or PCa metabolism.

Data extraction focused on molecular and metabolic determinants of AR-driven PCa biology, including AR expression and nuclear translocation, AR splice variants (e.g., AR-V7), and key metabolic pathways such as lipogenesis, mitochondrial biogenesis and function, and reactive oxygen species (ROS)/redox homeostasis. Particular attention was given to nutrient-sensing signaling, including the insulin/IGF-1 axis, as well as therapeutic outcomes, including response or resistance to ADT and AR-targeted agents. In parallel, data were collected on the systemic and tumor-specific effects of IF or caloric restriction, encompassing alterations in circulating metabolic hormones, glucose and lipid metabolism, body composition, treatment tolerance, and potential synergistic or sensitizing effects in combination with AR-directed therapies. The collected evidence was synthesized qualitatively to construct a mechanistic framework linking intermittent fasting with AR signaling and metabolic adaptations in PCa.

## 3. Androgen Receptor as a Metabolic Sensor

The AR is not merely a transcription factor governing androgen responsive gene expression but also functions as a central metabolic sensor that orchestrates multiple biochemical networks crucial for PCa cell survival and progression. In addition to its canonical role in regulating cell proliferation and differentiation, AR signaling has been consistently shown to influence key metabolic pathways, including lipid synthesis, mitochondrial function, and redox balance, thereby integrating hormonal cues with cellular energy and biosynthetic demands [11,18,19,20].

### 3.1. Lipogenesis

Androgen signaling directly induces the expression of a cadre of lipogenic enzymes, including fatty acid synthase (FASN), acetyl CoA carboxylase alpha (ACACA), and stearoyl-CoA desaturase 1 (SCD1), facilitating de novo fatty acid synthesis that supports the proliferative and survival needs of PCa cells [21,22,23].

Mechanistically, AR promotes lipid biosynthesis not only by direct transcriptional regulation of these enzymes but also by positively modulating sterol regulatory element binding protein-1 (SREBP-1), a master regulator of fatty acid and cholesterol biosynthesis, which further augments the expression of FASN and SCD1 [24]. This lipogenic program serves multiple functions: it provides energy storage reservoirs, generates lipid signaling molecules, and supplies structural components required for membrane synthesis, which is essential for rapid proliferation and may also influence AR nuclear localization and activity indirectly via membrane composition changes [21,23,24].

### 3.2. Mitochondrial Biogenesis

Androgen-mediated activation of AMP-activated protein kinase (AMPK) and downstream peroxisome proliferator-activated receptor gamma coactivator 1-alpha (PGC-1α) supports mitochondrial biogenesis and facilitates efficient adenosine triphosphate (ATP) production and redox balance, enabling PCa cells to meet the high bioenergetic and biosynthetic demands imposed by continuous growth stimuli [3]. Increased mitochondrial capacity also confers adaptive resilience to metabolic stress and may promote therapeutic resistance, as cells with enhanced oxidative metabolism can better sustain energy production under nutrient limiting conditions [25,26].

### 3.3. Redox Balance

Closely tied to mitochondrial function is the regulation of cellular redox homeostasis. AR activity contributes to redox balance by modulating antioxidant enzyme expression and influencing pathways such as the pentose phosphate pathway that generate reducing equivalents (e.g., nicotinamide adenine dinucleotide phosphate (NADPH)) necessary for ROS detoxification. This regulatory axis allows tumor cells to tolerate increased ROS production associated with rapid proliferation and androgen-driven metabolism, safeguarding genomic integrity and cellular survival.

Collectively, these findings establish AR as an integrated metabolic sensor that coordinates anabolic and catabolic pathways to sustain the growth, survival, and adaptability of PCa cells in changing metabolic environments [27,28].

## 4. Dietary Fasting Regimens as Modulators of Androgen Receptor Signaling in Prostate Cancer

Dietary energy restriction strategies have gained substantial interest as metabolic interventions capable of modulating tumor growth and therapeutic responsiveness across multiple malignancies. In PCa, where AR signaling is the principal driver of tumor proliferation and progression, interventions that alter systemic metabolism—such as reductions in insulin, IGF-1, and amino acid availability—may influence AR activity and downstream oncogenic programs. Four commonly investigated regimens include time-restricted eating (TRE), alternate-day fasting, fasting-mimicking diets, and chronic caloric restriction (CR). Collectively, these approaches converge on shared metabolic axes implicated in anabolic hormone regulation and nutrient sensing pathways, offering opportunities to modulate AR signaling and potentially enhance therapeutic responses.

Time-restricted eating involves confining daily food intake to a specified window (e.g., 6–12 h) without deliberate caloric reduction, thereby producing prolonged fasting intervals that shift cellular metabolism toward fatty-acid oxidation and improve insulin sensitivity. While TRE has been primarily studied for its beneficial effects on circadian rhythm, metabolic risk factors, and systemic inflammation, mechanistic evidence linking TRE directly to AR regulation in PCa remains limited. Observational studies report associations between extended nightly fasting durations (≥13 h) and reduced recurrence risk in diverse cancer populations, suggesting that fasting intervals may influence hormone-related pathways and endocrine milieu, although direct interrogation of AR signaling in PCa cohorts is pending [29].

Alternate-day fasting alternates days of severe caloric restriction (~0–25% of energy requirements) with ad libitum feeding and has provided the strongest mechanistic evidence for direct modulation of AR in preclinical PCa models. In recent work, ADF induced amino-acid deprivation that impaired AR mRNA translation through translational stress mechanisms, resulting in significant reductions in AR protein levels and AR signaling activity. This metabolic suppression of AR concomitantly enhanced the efficacy of the AR antagonist enzalutamide in vivo, producing greater tumor regression than pharmacologic therapy alone [18]. These findings establish nutrient availability as a previously underappreciated regulator of AR protein homeostasis and support the hypothesis that metabolic interventions such as ADF can act as non-pharmacologic modulators of AR signaling with potential to delay therapeutic resistance.

Fasting-mimicking diets are structured periodic interventions in which low overall calories and specific macronutrient composition are employed over short cycles (typically 3–5 days per month) to elicit fasting-like metabolic responses while maintaining limited nutrient intake. FMD consistently reduces circulating glucose, insulin, and IGF-1 levels and suppresses downstream mechanistic target of rapamycin (mTOR) signaling in both preclinical and clinical settings, effects which intersect with key growth factor pathways associated with oncogenesis. In animal models, such metabolic reprogramming enhances immune cytotoxicity and suppresses tumor progression [30]. In early clinical implementations, periodic FMD cycles resulted in improvements in metabolic risk profiles and markers associated with systemic inflammation in cancer patients undergoing standard therapies [31]. Although direct mechanistic studies linking FMD to AR dynamics in PCa are still forthcoming, reductions in systemic IGF-1 and insulin signaling—with known crosstalk to steroid receptor pathways—provide an indirect rationale for potential modulation of AR activity.

Chronic caloric restriction involves sustained reduction in daily energy intake (typically 20–40%) without malnutrition and is among the most widely studied dietary interventions in cancer biology. Preclinical evidence demonstrates that CR delays tumor initiation, suppresses proliferation, and alters multiple anabolic and inflammatory signaling pathways implicated in cancer progression. While CR studies rarely measure AR signaling as a primary endpoint, the metabolic milieu created by chronic energy restriction—characterized by decreased circulating hormones, growth factors, and nutrient signals—overlaps with pathways known to regulate AR transcriptional activity and protein synthesis. Long-term CR consistently lowers IGF-1, insulin, and mTOR activity—drivers of growth and proliferation that have documented interactions with steroid receptor signaling networks [32].

Collectively, fasting-based interventions exert overlapping metabolic effects that may influence androgen receptor biology in PCa. Among these regimens, ADF currently provides the most compelling mechanistic evidence for direct AR modulation via nutrient limitation and translational stress, suggesting metabolic restriction as a viable strategy to enhance sensitivity to androgen-targeted therapy. TRE offers metabolic and circadian benefits with emerging evidence of hormetic effects, while FMD achieves favorable systemic metabolic remodeling with ongoing clinical evaluation. Chronic CR, although challenging to implement long term in humans, presents robust systemic effects that overlap with pathways relevant to AR regulation. Integrating these dietary strategies into PCa management holds promise as an adjunct to conventional therapies, but prospective clinical trials with AR-specific endpoints are necessary to validate their efficacy, optimize regimen parameters, and clarify their role in delaying resistance to androgen receptor signaling inhibitors.

For the purposes of this review, we focus on dietary interventions that fall within the definition of IF, including TRE, ADF, and FMD, which are characterized by repeated periods of voluntary nutrient deprivation interspersed with periods of normal intake.

### Intermittent Fasting and Insulin/Insulin-like Growth Factor 1 Signaling

IF exerts systemic metabolic effects that extend beyond simple caloric reduction, significantly impacting the insulin/IGF 1 axis, a major regulator of growth signaling and cellular metabolism in cancer [33,34,35]. In multiple preclinical and clinical contexts, IF and FMD have been shown to reduce circulating insulin and IGF 1 levels, which in turn attenuates downstream pathways such as phosphoinositide 3-kinase (PI3K)/v-akt murine thymoma viral oncogene homolog (AKT)/mechanistic target of rapamycin (mTOR), frequently dysregulated in PCa and associated with metabolic adaptation and treatment resistance, as reviewed by Vernieri et al. [36].

Decreased insulin/IGF-1 signaling reduces PI3K/AKT/mTOR activity, blunting anabolic signaling and proliferation, which potentially may synergize with ADT to constrain tumor growth [37,38,39,40]. Fasting also enhances metabolic stress signaling through activation of AMPK and sirtuin pathways, contributing to improved mitochondrial efficiency and cellular stress responses [15,41]. Furthermore, IF-induced hormonal changes can potentially modulate AR coactivators and epigenetic regulators through altered chromatin accessibility and metabolic cofactor availability [42,43,44,45,46], suggesting that IF may act as a physiological signal capable of modulating growth and metabolic pathways that intersect with AR signaling in PCa.

## 5. Crosstalk Between Intermittent Fasting and Androgen Receptor Signaling

IF and other forms of caloric restriction induce profound metabolic stress in tumor cells, which engages nutrient-sensing pathways such as AMPK, mTOR, and stress kinase signaling [47,48,49]. These metabolic shifts can alter AR signaling in PCa, a pathway central to disease progression and therapeutic response [50,51,52,53]. Emerging evidence shows that metabolic stress from IF modulates AR expression and activity, including AR nuclear translocation and influence on AR splice variants such as AR-V7 [18,54]. These interactions suggest that IF may sensitize PCa cells to ADT and AR-targeted agents by disrupting AR signaling at multiple regulatory levels [18]. Constitutively active AR splice variants such as AR-V7 retain nuclear localization independently of ligand binding, promoting transcriptional activity in castration-resistant prostate cancer (CRPC) [55,56,57,58,59]. IF-induced nutrient deprivation may modulate RNA splicing factors (e.g., serine and arginine rich splicing factor 1 (SRSF1)) and alter AR-V7 transcriptional programs, potentially increasing therapeutic vulnerability [18,60].

### 5.1. Androgen Receptor Nuclear Translocation

The canonical mechanism of AR activation involves androgen binding to the ligand-binding domain (LBD), dissociation from heat-shock proteins, receptor dimerization, and translocation into the nucleus, where AR regulates target gene expression by binding androgen response elements (AREs) in DNA [45,61]. Post-translational modifications (PTMs) such as phosphorylation, acetylation, ubiquitination, and SUMOylation of AR also influence its stability, subcellular localization, and transcriptional activity [46,61]. For example, acetylation of lysine residues by coactivators such as lysine acetyltransferase 5 (KAT5) (historically Tat-interacting protein of 60 kDa (Tip60)) promotes release of AR from chaperones, facilitating nuclear entry, whereas deacetylation by enzymes such as sirtuin 1 (SIRT1) represses AR activity [45].

Intermittent fasting or caloric restriction imposes metabolic stress by reducing circulating nutrients (e.g., amino acids, glucose) and activating stress response pathways, including AMPK and p38 mitogen-activated protein kinase (p38 MAPK) [49,62,63,64,65]. In preclinical PCa models, ADF significantly lowers intratumoral amino acid levels, resulting in reduced global protein synthesis and selective impairment of AR protein translation [18,54]. Reduced AR protein availability is expected to indirectly limit the efficiency of AR nuclear translocation, as substrate levels for nuclear import are decreased and post-translational modifications (PTM) landscapes are altered under nutrient stress conditions. Although direct experimental assessments of AR nuclear translocation under IF are limited, attenuation of AR abundance and signaling provides a mechanistic explanation for the observed increase in sensitivity to AR antagonists such as enzalutamide [18,54].

Furthermore, metabolic stress induced by IF activates kinases such as p38 MAPK and general control nonderepressible 2 (GCN2), which regulate translational checkpoints and can influence chaperone availability, AR phosphorylation status, and nuclear transport machinery [18,54]. Stress-activated kinase signaling has been shown to modulate nuclear transport components in multiple cellular systems [66,67,68,69], suggesting that IF-associated stress may alter AR localization dynamics in PCa cells [70,71]. Collectively, these mechanisms link IF-induced metabolic stress to altered AR PTMs and reduced nuclear translocation efficiency, thereby sensitizing PCa cells to ADT and AR-targeted therapies [18,54].

Table 1 summarizes the key mechanisms through which IF-induced metabolic stress can influence AR nuclear translocation in PCa. The table integrates upstream metabolic cues, stress-activated signaling pathways, and post-translational modifications that collectively modulate AR availability, nuclear import efficiency, and transcriptional activity, providing a concise framework for mechanistic interpretation and translational biomarker selection.

### 5.2. Androgen Receptor Splice Variants (Androgen Receptor Splice Variant 7)

One of the dominant mechanisms of resistance to ADT and AR-targeted agents in advanced PCa is the expression of constitutively active AR splice variants lacking the LBD, most notably AR-V7 [55,58]. AR-V7 retains the N-terminal transactivation domain and DNA-binding domain, enabling ligand-independent nuclear localization and transcriptional activity characteristic of CRPC [55,59]. Clinical studies have demonstrated that detection of AR-V7 in circulating tumor cells is associated with resistance to enzalutamide and abiraterone, underscoring its clinical significance [56].

Generation of AR splice variants in PCa is influenced by transcriptional dynamics and recruitment of specific RNA splicing factors, including U2 snRNP auxiliary factor 65 kDa subunit (U2AF65) and alternative splicing factor 2, known as SRSF1 (historically alternative splicing factor/splicing factor 2 (ASF/SF2)), to AR pre-mRNA; these processes are modulated by AR transcriptional activity and RNA polymerase II elongation rates [58,78]. Metabolic stress, such as nutrient deprivation induced by IF, broadly affects cellular RNA processing pathways and splicing factor activity in multiple cancer models, where stress conditions have been shown to alter alternative splicing decisions. Although direct experimental studies linking IF to AR-V7 splicing are currently lacking, IF-induced metabolic stress may plausibly modulate the activity or phosphorylation status of splicing factors involved in AR variant generation. Notably, the activity of SR proteins such as SRSF1 is regulated by phosphorylation through nutrient- and stress-sensitive kinase pathways [79].

Once expressed, AR-V7 exhibits efficient nuclear localization independent of ligand binding, likely mediated by alternative nuclear localization signals present in its truncated structure [57,59]. This constitutive nuclear presence enables persistent transcription of AR target genes, including those involved in DNA repair, cell survival, and metabolic adaptation, thereby promoting resistance to AR-targeted therapies [57,80]. IF may further influence AR-V7 activity by altering coactivator availability and nutrient-sensing pathways that converge on nuclear transcriptional complexes. Although direct mechanistic evidence remains limited, the metabolic context created by IF—characterized by reduced amino acid availability and sustained stress signaling—could reshape the AR-V7 nuclear interactome or downstream transcriptional programs, potentially enhancing vulnerability to combination therapeutic strategies. To summarize potential mechanisms linking intermittent fasting (IF) to AR-V7 regulation, we provide Table 2, which outlines key regulatory axes through which IF-induced metabolic stress may influence splice variant expression, nuclear localization, and downstream transcriptional programs. The table integrates proposed IF-related effects with expected impacts on AR-V7 and supporting mechanistic evidence from relevant studies.

### 5.3. Metabolic Reprogramming of Androgen Receptor Signaling Under Intermittent Fasting

Androgen receptor signaling is tightly coupled to cellular metabolism, as AR directly regulates the transcription of genes involved in glucose uptake, lipid synthesis, mitochondrial function, and amino acid metabolism in PCa cells [83]. Conversely, metabolic pathways exert feedback control over AR activity, creating a bidirectional regulatory network. IF induces systemic and intratumoral metabolic changes—most notably reduced glucose and amino acid availability—that reprogram AR-driven transcriptional programs in PCa.

AR activation promotes anabolic metabolism, including de novo lipogenesis through transcriptional induction of FASN, ACACA, and sterol regulatory element binding transcription factor 1 (SREBF1) [83]. These pathways are energetically demanding and depend on adequate nutrient supply. Under conditions of caloric restriction or fasting, activation of AMPK suppresses anabolic processes and antagonizes mTORC1 signaling, a key regulator of protein synthesis and AR-driven metabolic output [84,85,86]. Experimental studies in PCa models demonstrate that AMPK activation suppresses AR transcriptional activity independently of androgen levels, linking energy stress directly to reduced AR output [50].

Recent in vivo work using ADF showed that nutrient limitation selectively disrupts AR-regulated metabolic gene expression while sparing housekeeping pathways, suggesting that AR-driven anabolic programs are particularly vulnerable to metabolic stress [18]. This metabolic reprogramming results in reduced lipid synthesis, impaired mitochondrial respiration, and diminished redox capacity, collectively constraining tumor growth. Importantly, these effects occur even in the presence of residual AR signaling, indicating that IF shifts the qualitative nature of AR transcription rather than merely suppressing receptor abundance [14,18,54].

Additionally, AR regulates amino acid transporters and one-carbon metabolism genes that support protein synthesis and nucleotide biosynthesis [87,88]. IF-induced amino acid depletion activates the general control nonderepressible 2 (GCN2)–eukaryotic translation initiation factor 2 subunit alphae (eIF2α) pathway, leading to translational repression and altered transcriptional feedback onto AR signaling networks [89,90,91,92,93]. Together, these findings support a model in which IF reshapes AR signaling by imposing metabolic constraints that selectively blunt AR-dependent anabolic and biosynthetic programs critical for PCa progression. Importantly, it remains essential to distinguish fasting-specific effects from those attributable to overall caloric deficit or weight loss. Many metabolic and hormonal shifts described above—including suppression of mTOR signaling, modulation of redox balance, and alterations in AR-regulated anabolic transcriptional programs—may partially reflect negative energy balance rather than fasting periodicity per se. Carefully controlled experimental designs incorporating isocaloric fasting protocols or matched weight-loss comparator groups will therefore be required to isolate fasting-dependent mechanisms from general energy restriction effects.

**Figure 1:**
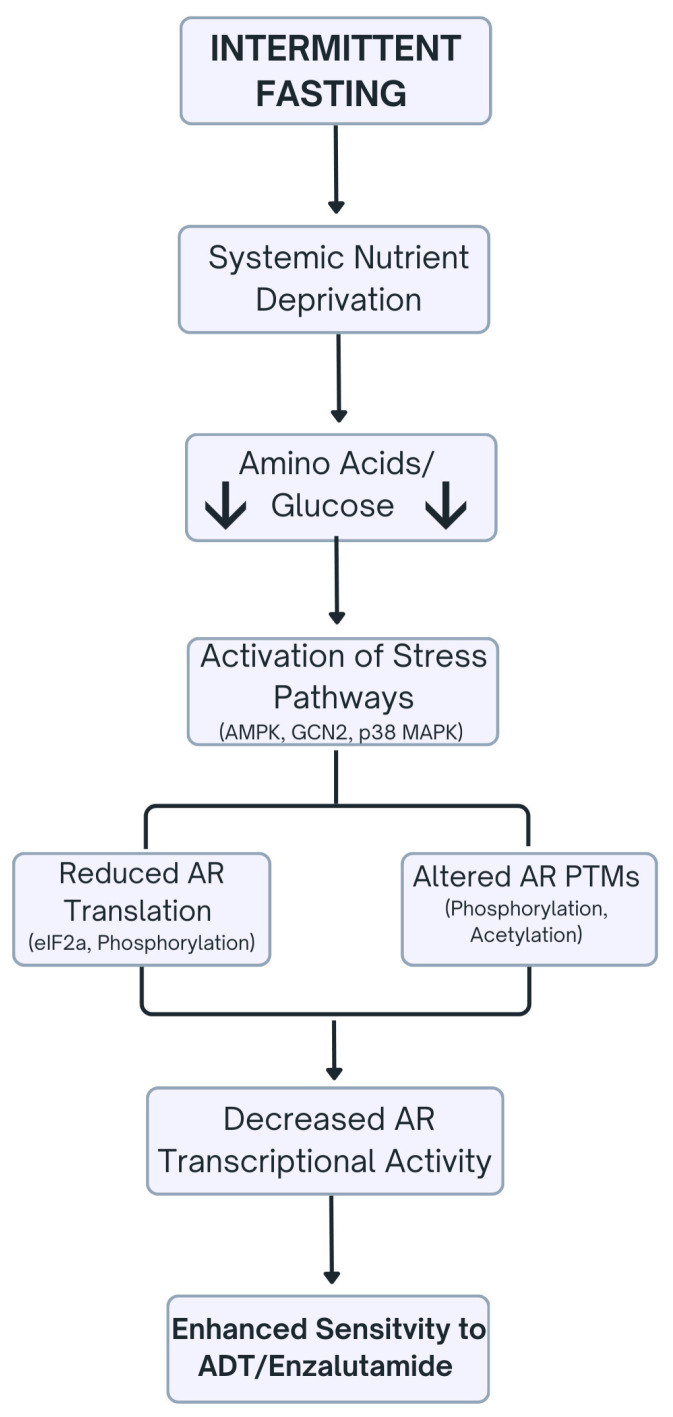
Schematic overview of the effects of IF on AR signaling in prostate cancer. Intermittent fasting induces systemic nutrient deprivation, resulting in decreased availability of key metabolites such as amino acids and glucose. This metabolic stress activates intracellular stress-response pathways, including AMPK, GCN2, and p38 MAPK. These pathways converge on the AR axis through two complementary mechanisms: (i) suppression of AR translation via eIF2α phosphorylation, leading to a reduced cytoplasmic AR pool, and (ii) modulation of AR PTMs, including phosphorylation and acetylation, which impair AR nuclear import. Collectively, these effects diminish AR transcriptional activity, thereby enhancing the sensitivity of prostate cancer cells to ADT and AR-targeting agents such as enzalutamide.

Figure 1 provides a schematic overview of the molecular mechanisms by which IF modulates AR signaling in PCa, highlighting nutrient deprivation-induced stress pathways that suppress AR translation, nuclear localization, and transcriptional activity.

**Figure 2:**
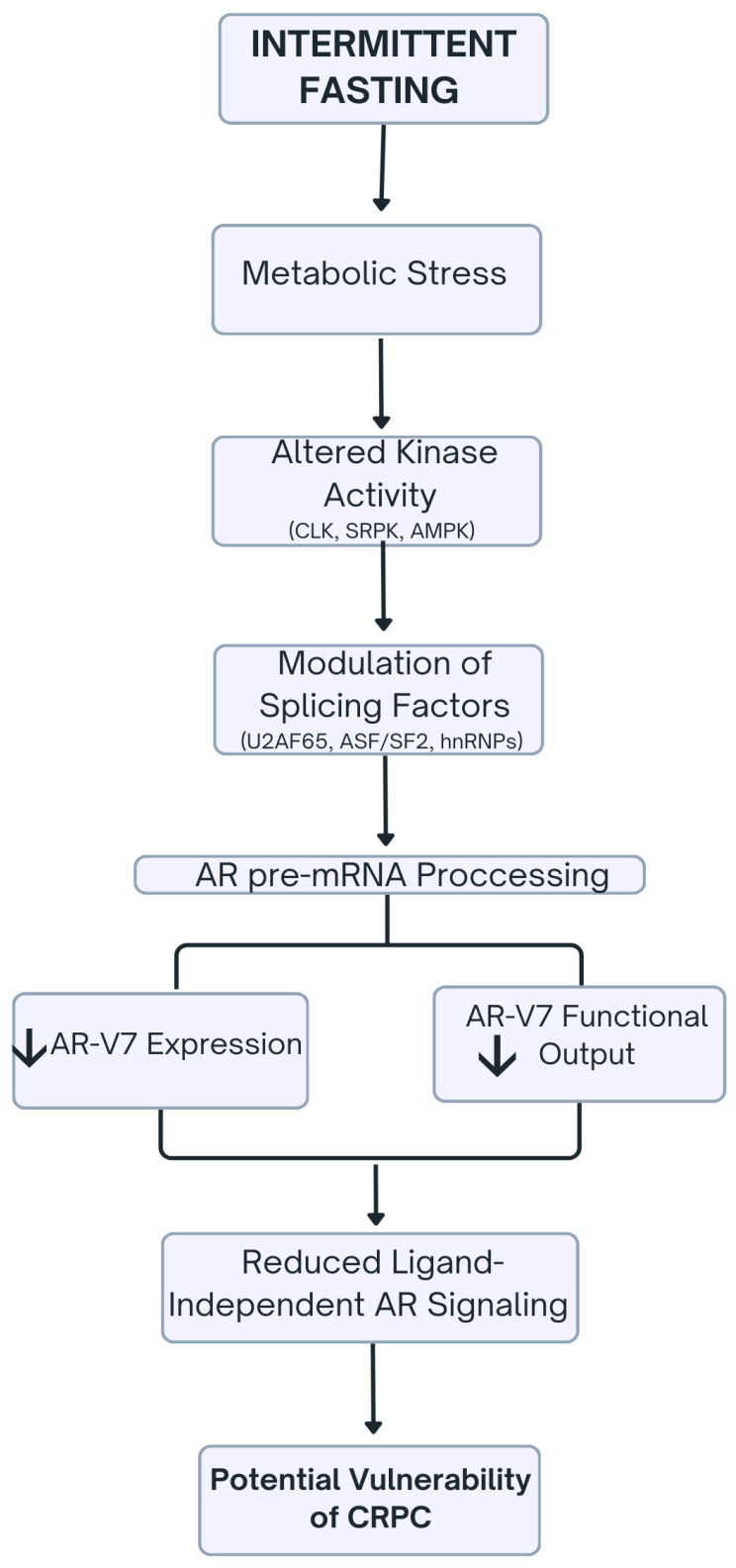
Schematic representation of intermittent fasting effects on AR splicing and AR-V7 signaling in prostate cancer. Intermittent fasting induces metabolic stress, which leads to altered activity of key kinases such as CDC-like kinase (CLK), serine-arginine protein kinase (SRPK), and AMPK. These kinases modulate splicing factor function, including U2AF65, ASF/SF2, and heterogeneous nuclear ribonucleoprotein (hnRNP) proteins, resulting in altered AR pre-mRNA processing. Consequently, the expression or functional output of the splice variant AR-V7 is reduced, diminishing ligand-independent AR signaling. This mechanism may expose CRPC cells to potential vulnerabilities that could be therapeutically exploited.

Figure 2 illustrates the proposed effects of IF on AR splicing, emphasizing the regulation of splicing kinases and factors that influence AR-V7 expression and ligand-independent AR signaling.

## 6. Intermittent Fasting as a Modulator of Therapeutic Response in Androgen Receptor-Driven Prostate Cancer

The biological and therapeutic implications of IF in PCa are context dependent. In localized disease, AR signaling is generally intact, and early resistance is driven by metabolic adaptations and emerging androgen-independent clones. In metastatic hormone-sensitive prostate cancer (mHSPC), IF may complement androgen ADT by delaying castration-resistant mechanisms. In metastatic castration-resistant prostate cancer (mCRPC), adaptive resistance is dominated by AR overexpression, AR splice variants (notably AR-V7), and metabolic rewiring [56,80]. Therefore, mechanistic and biomarker interpretations should be stratified by disease stage.

Preclinical studies demonstrate that FMD enhance antitumor efficacy of AR antagonists in PCa models [18,94]. ADF synergizes with enzalutamide by reducing AR protein synthesis and downstream transcriptional output, delaying tumor progression compared with either intervention alone [18]. These effects are accompanied by suppression of mTORC1 signaling, pathway implicated in resistance to AR-targeted therapy. Suppression of mTORC1 signaling has been implicated in overcoming compensatory activation of alternative survival pathways following AR-targeted therapy, as PI3K/AKT/mTOR signaling becomes hyperactivated in response to AR blockade and can facilitate therapeutic resistance. Reciprocal feedback between AR and mTOR pathways supports the rationale for combined AR and mTOR inhibition to delay resistance development [95,96,97]. 

Metabolic stress induced by IF may also limit the emergence of castration-resistant phenotypes by constraining adaptive metabolic pathways required for survival under androgen-depleted conditions. CRPC cells exhibit increased reliance on oxidative phosphorylation, lipid oxidation, and amino acid scavenging pathways [98,99], all of which are sensitive to nutrient availability. Experimental evidence indicates that dietary restriction can suppress these metabolic adaptations, thereby enhancing vulnerability to systemic therapy [94,100,101,102,103,104]. 

While direct clinical outcome data on intermittent fasting combined with ADT remain limited, ongoing translational and clinical research (e.g., pilot study NCT06172283) suggests that dietary energy restriction strategies may be feasible and could influence treatment tolerance and toxicity profiles in prostate cancer patients receiving ADT [105]. 

However, energy deficit, weight loss, and body composition changes may contribute to observed biomarker shifts; therefore, clinical studies should include matched controls and monitor lean mass to isolate fasting-specific effects.

**Figure 3:**
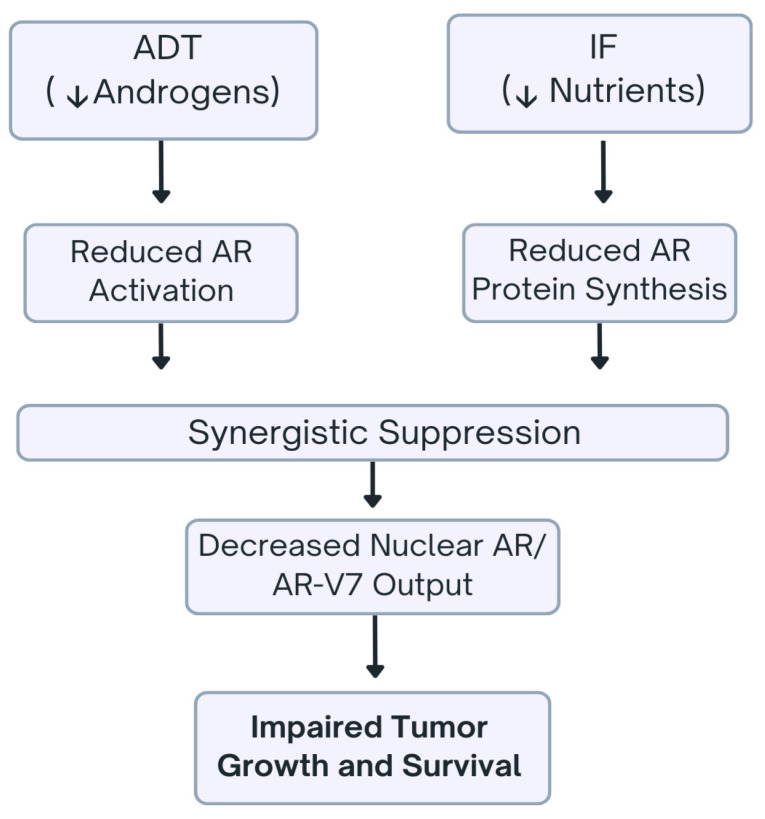
Synergistic suppression of androgen receptor signaling by androgen deprivation therapy and intermittent fasting in prostate cancer. This schematic illustrates the combined impact of ADT and IF on AR signaling in prostate cancer. ADT, through reduction in circulating androgens, diminishes AR activation, while IF limits nutrient availability, leading to reduced AR protein synthesis. These parallel effects converge in a synergistic suppression of AR signaling, resulting in decreased nuclear AR and AR-V7 output. Consequently, tumor cell growth and survival are impaired, highlighting a potential combinatorial therapeutic strategy to enhance prostate cancer treatment efficacy.

Figure 3 depicts synergistic suppression of AR signaling by combined ADT and IF, showing convergence of hormonal and metabolic interventions to reduce AR and AR-V7 activity in PCa cells.

## 7. Clinical Context and Translational Implications Across Disease Stages

The biological and clinical relevance of IF is likely to differ across stages of PCa, necessitating stage-specific translational considerations. In localized PCa, where disease progression remains predominantly androgen-dependent and tumor burden is limited [106], IF may primarily function as a metabolic modifier. In this setting, potential benefits may include modulation of insulin–IGF-1 signaling and attenuation of anabolic pathways such as mTOR, which are sensitive to fasting and caloric restriction [30,107]. These systemic changes may be particularly relevant in patients undergoing radiotherapy or early ADT, where metabolic health influences long-term outcomes [108]. Here, IF may serve as a disease-modifying adjunct rather than a resistance-targeting strategy.

In metastatic hormone-sensitive PCa, where tumors remain responsive to ADT but exhibit higher systemic tumor burden and metabolic demand [109], IF may exert both systemic and tumor-intrinsic effects. The combination of androgen suppression with fasting-induced metabolic stress could enhance AMPK activation and suppress mTOR signaling, pathways consistently modulated during fasting or fasting-mimicking diets in vivo [30,94]. However, ADT is associated with adverse metabolic sequelae, including sarcopenia, increased adiposity, and insulin resistance [108,110,111], underscoring the need for structured nutritional and resistance exercise co-interventions in this population.

In contrast, mCRPC is characterized by AR pathway reactivation, AR splice variants (e.g., AR-V7), and profound metabolic plasticity [58,80]. AR-V7 has been directly associated with resistance to enzalutamide and abiraterone in clinical cohorts [56]. Given the central role of metabolic rewiring and stress adaptation in mCRPC progression [80], IF may have greater mechanistic relevance as a metabolic stress-amplifying strategy. However, patients with advanced disease frequently exhibit frailty, treatment-related toxicity, and body composition deterioration [108,110,111], which may limit tolerance to prolonged caloric restriction. Short-cycle or fasting-mimicking regimens, as evaluated in clinical oncology settings [49,112], may therefore represent more feasible approaches than sustained caloric restriction.

Importantly, despite compelling mechanistic rationale and supportive preclinical evidence linking fasting to IGF-1 suppression, AMPK activation, and mTOR inhibition [16,49,94], robust stage-stratified clinical trials evaluating IF in PCa remain scarce. Future prospective studies should incorporate: (i) stratification by disease stage (localized, mHSPC, mCRPC), (ii) standardized IF regimens (e.g., time-restricted eating vs. fasting-mimicking diet), (iii) integrated biomarker panels capturing AR signaling, metabolic adaptation, and redox status, and (iv) longitudinal monitoring of body composition using validated modalities such as dual-energy X-ray absorptiometry (DEXA). Given its non-pharmacological nature, low cost, and favorable safety profile in other clinical populations [112], IF represents a feasible and potentially scalable adjunct to androgen-targeted therapy. However, its implementation in PCa must be evidence-driven, stage-specific, and integrated with mitigation strategies to prevent sarcopenia and treatment-related frailty.

## 8. Translational Relevance and Rationale for Biomarker Selection

Successful clinical translation of IF in PCa requires biomarkers capturing tumor-intrinsic AR signaling and systemic metabolic adaptations. Biomarkers should reflect the following:

- AR abundance, localization, and transcriptional activity;
- Nutrient-sensing pathways, metabolic rewiring, mitochondrial adaptation, and redox homeostasis.

Ideal biomarkers should be biologically relevant to AR–metabolism crosstalk, feasible in tissue or liquid biopsies, and predictive of therapeutic response. Integration of tumor-based and systemic metabolic biomarkers enables patient stratification and rational combination strategies with ADT or AR-targeted agents, stratified by disease stage and regimen type (TRE, ADF, FMD). The following sections outline candidate biomarkers organized by functional domains relevant to IF–AR interactions and PCa therapy resistance. Table 3 summarizes the global translational impact of IF regimens in PCa, integrating directional changes in key metabolic, signaling, and oxidative stress biomarkers with in vivo and clinical evidence. The table highlights how IF, including TRE, ADF, and FMD, modulates tumor-relevant pathways such as AMPK activation, mTOR inhibition, and IGF-1/insulin reduction, which collectively converge on androgen receptor signaling and cancer metabolism. In addition, IF promotes adaptive responses including enhanced mitochondrial function, fatty acid oxidation (FAO), autophagy, and antioxidant defenses, while improving systemic metabolic parameters such as serum insulin, ketone bodies, and body composition. By integrating both preclinical and clinical findings, this table provides a concise overview of the mechanistic and translational effects of IF in PCa models, supporting its potential as an adjunctive therapeutic strategy.

### 8.1. Biomarkers of AR Signaling and Activity

Table 4 summarizes tissue- and blood-based biomarkers of AR abundance, localization, transcriptional output, and therapy resistance mechanisms. Applicability by disease stage is indicated.

### 8.2. Metabolic and Nutrient-Sensing Biomarkers

These biomarkers capture systemic and tumor-specific metabolic responses to fasting and energy restriction. Table 5 outlines biomarkers reflecting energy stress signaling and anabolic pathway suppression.

### 8.3. Mitochondrial and Bioenergetic Biomarkers

Mitochondrial adaptation is central to metabolic flexibility and stress tolerance under IF. Table 6 presents biomarkers of mitochondrial adaptation, remodeling, and enhanced oxidative capacity under fasting-induced stress.

### 8.4. Redox Homeostasis and Oxidative Stress Biomarkers

These markers reflect the balance between ROS production and antioxidant defense, a key determinant of therapy sensitivity. Table 7 lists biomarkers reflecting ROS balance and antioxidant defense mechanisms:

### 8.5. Biomarkers of Therapeutic Response and Resistance

Biomarkers reflecting AR signaling provide direct insight into the biological efficacy of IF in modulating the androgen axis. Table 8 summarizes proliferation, apoptosis, DNA damage, and tumor burden markers, stratified by disease stage and fasting regimen type.

### 8.6. Perspective

Together, these biomarkers form a multidimensional framework for assessing the biological and clinical impact of intermittent fasting in PCa. By integrating AR signaling metrics with metabolic, mitochondrial, and redox readouts, this approach enables mechanistic stratification of patients and provides a rational basis for designing biomarker-driven clinical trials combining IF with AR-targeted therapies, particularly in CRPC.

## 9. Challenges and Future Directions

From a clinical perspective, PCa represents a major global health burden, ranking second in incidence and fifth in cancer-related mortality among men worldwide [136]. Unlike novel pharmacologic agents, IF is a non-invasive, low-cost, and potentially scalable intervention that does not require new drug development or long-term toxicity profiling. Restricting food intake to a defined daily window (e.g., a 16:8 schedule) could be feasibly integrated alongside standard-of-care therapies such as ADT, AR-targeted agents, or chemotherapy. However, clinical enthusiasm must be tempered by rigorous evaluation.

Accordingly, well-designed prospective clinical trials are urgently needed to determine whether IF can modulate systemic metabolism, intratumoral AR activity, splice variant expression, and clinically meaningful endpoints in PCa patients. Such trials should incorporate comprehensive biomarker assessment—including metabolic parameters, AR nuclear localization, AR-V7 status, mitochondrial and redox signatures—and stratify patients by disease stage. Equally important are evaluations of adherence, safety, body composition changes, and quality-of-life metrics, particularly in vulnerable populations.

Taken together, advancing IF from mechanistic promise to clinical application will require coordinated preclinical, translational, and clinical efforts. A stage-specific and regimen-specific framework, coupled with robust biomarker-driven trial design, will be essential to define whether metabolic stress induced by IF can meaningfully enhance therapeutic responsiveness in AR-driven PCa. Only through such rigorous evaluation can evidence-based recommendations be formulated and responsibly implemented in clinical practice.

The biological and therapeutic implications of IF in PCa are context-dependent and should be interpreted within the framework of disease stage. In localized PCa, AR signaling is generally intact, and primary resistance mechanisms involve early metabolic adaptations and the emergence of androgen-independent subclones [121]. IF may primarily act as a metabolic modifier in this setting, modulating insulin–IGF-1 signaling, AMPK activation, and mTOR suppression, potentially enhancing the response to radiotherapy or early ADT without targeting resistance mechanisms directly [137,138].

In mHSPC, where tumors remain responsive to ADT but exhibit higher systemic tumor burden, IF may complement androgen suppression by imposing metabolic stress that modulates AR activity and delays castration-resistant adaptations [16,18]. Systemic effects may include enhanced AMPK activation, mTOR inhibition, and favorable circadian alignment of feeding windows [139]. However, ADT in this population is associated with sarcopenia, fat gain, and insulin resistance, highlighting the need for nutritional guidance, structured resistance exercise, and monitoring of lean mass [110,140].

In mCRPC, adaptive resistance is frequently dominated by AR overexpression, constitutively active splice variants (notably AR-V7), and profound metabolic rewiring [137,141]. AR-V7 has been associated with resistance to enzalutamide and abiraterone in clinical cohorts [137]. In this setting, IF may serve as a metabolic stress-amplifying strategy, but patient frailty, body composition deterioration, and treatment-related toxicity may limit tolerability. Short-cycle or fasting-mimicking interventions may be more feasible than sustained caloric restriction.

IF is not a biologically uniform intervention. The term encompasses multiple regimens, including TRE, ADF, and FMD. These regimens differ in fasting duration, caloric targets, macronutrient composition, and frequency, producing distinct metabolic effects. For instance, prolonged fasting or FMD robustly suppresses circulating IGF-1 and amino acid availability, whereas TRE primarily affects circadian alignment and insulin dynamics. Differential regulation of metabolic pathways—including IGF-1/PI3K–AKT–mTOR, AMPK, ketogenesis, mitochondrial remodeling, and nutrient-sensing networks—should therefore be considered when interpreting preclinical or clinical results [16,138,142].

## 10. Discussion

The accumulated evidence suggests a compelling link between metabolic state and AR signaling in PCa, wherein metabolic interventions such as IF can modulate tumor biology and therapeutic response. Preclinical models of PCa have demonstrated that ADF reduces amino acid availability within tumors, leading to decreased AR mRNA translation and enhanced sensitivity to AR antagonists such as enzalutamide [18,143]. These results underscore the notion that metabolic stress induced by IF can directly influence AR signaling at the level of protein synthesis, thereby potentially improving the efficacy of anti-androgen therapy.

IF also exerts broader effects on systemic metabolic regulators, notably insulin and IGF-1, which are known to interact with oncogenic pathways including PI3K/AKT/mTOR [14,144]. While clinical data remain mixed regarding the impact of dietary manipulation on IGF-1 levels—such as the observation that isocaloric protein restriction alone does not significantly reduce IGF-1 in men with localized PCa [145]—preclinical and epidemiological evidence supports the concept that energy restriction and fasting regimens can lower circulating IGF-1 and modulate downstream signaling that interacts with AR pathways [14,144]. Indeed, IGF-1 signaling has been implicated in both androgen-dependent and androgen-independent PCa progression, suggesting that its modulation may have therapeutic relevance [146,147].

Beyond hormonal effects, IF influences cellular energy sensors such as AMPK and mTOR, which integrate nutrient cues with growth and survival pathways [49,148]. Activation of AMPK under fasting conditions promotes a shift toward catabolic metabolism and can antagonize mTORC1 signaling, potentially blunting anabolic processes that support tumor growth. Although direct evidence of IF-induced AMPK activation in PCa models remains to be fully characterized, the established role of AMPK–peroxisome proliferator-activated receptor gamma coactivator 1 alpha (PGC-1α) signaling in linking androgen signaling to metabolism supports an integrated model in which metabolic stress and hormonal cues converge to determine tumor behavior [149].

Despite encouraging preclinical insights, significant challenges remain. First, the translation of IF regimens from rodent models to human patients requires careful optimization, as differences in metabolism, tumor heterogeneity, and clinical tolerability may influence outcomes.

Another consideration is body composition. ADT and AR pathway inhibitors promote sarcopenia, fat gain, and frailty. IF may exacerbate lean mass loss if not carefully managed. Clinical translation should therefore integrate mitigation strategies including adequate protein intake, resistance exercise programs, and longitudinal assessment of muscle mass using DEXA or bioelectrical impedance analysis (BIA).

It is also important to distinguish the effects of fasting per se from those attributable to overall caloric deficit and weight loss. Many observed metabolic and hormonal changes—including reductions in circulating IGF-1, suppression of mTOR signaling, and alterations in redox balance—may partially reflect negative energy balance or changes in body composition rather than fasting timing alone. Future clinical and preclinical studies should therefore incorporate controlled designs, including isocaloric fasting protocols, matched weight-loss comparator groups, and systematic assessment of body composition, in order to isolate fasting-specific mechanisms from those driven by energy restriction.

Next, the complexity of metabolic and hormonal networks necessitates mechanistic studies to delineate how IF impacts AR splice variant expression, coactivator dynamics, and resistance phenotypes. Third, circadian rhythms and chrononutrition may modulate metabolic responses to fasting, further complicating experimental and clinical design.

Overall, the integration of IF into PCa therapy represents a promising yet nascent approach that warrants rigorous investigation in well controlled clinical studies, accompanied by mechanistic biomarker analyses to guide precision intervention strategies.

## 11. Conclusions

IF represents a promising metabolic intervention that can modulate AR signaling and tumor metabolism in PCa, offering potential to enhance sensitivity to AR-targeted therapies. Preclinical studies demonstrate that IF can reduce AR expression and signaling, and that energy restriction impacts systemic regulators such as insulin and IGF 1, which intersect with oncogenic pathways. However, clinical evidence remains preliminary and warrants further investigation to establish optimal fasting regimens, clarify mechanisms of action, and identify biomarkers predictive of response. Future studies should incorporate translational endpoints that capture AR activity, metabolic adaptations, and therapeutic outcomes to fully elucidate the potential of IF as an adjunctive strategy in the management of androgen-driven PCa.

## Figures and Tables

**Table 1:** Mechanisms Linking Intermittent Fasting-Induced Metabolic Stress with AR Nuclear Translocation in Prostate Cancer.

Level of Regulation	IF-Induced Change	Effect on AR Nuclear Translocation	Molecular Basis	Key References
AR protein abundance	Reduced amino acid availability	Decreased pool of cytoplasmic AR available for nuclear import	Inhibition of AR mRNA translation via GCN2–eIF2α pathway	[72]
Post-translational modifications	Altered phosphorylation/acetylation balance	Impaired dissociation from chaperones; reduced nuclear entry	AMPK and stress kinase signaling modulate AR PTMs and cofactor recruitment	[70]
Chaperone interaction	Stress-induced changes in HSP90 dynamics	Delayed AR activation and nuclear import	Metabolic stress alters HSP90 function and AR folding	[73,74]
Nuclear transport machinery	Reduced efficiency of importins	Slower AR nuclear accumulation	Energy stress impacts nuclear transport processes	[75,76]
Androgen signaling output	Lower transcriptional activity	Sensitization to ADT and AR antagonists	Reduced nuclear AR leads to weaker ARE-driven transcription	[77]

Abbreviations: eIF2α, eukaryotic initiation factor 2α; GCN2, general control nonderepressible 2; HSP90, heat shock protein 90.

**Table 2:** Potential Mechanisms by Which IF May Modulate AR Splice Variant (AR-V7) Expression and Activity.

Regulatory Axis	IF-Related Mechanism	Expected Impact on AR-V7	Supporting Evidence
*AR* gene transcription	Reduced transcriptional elongation	Lower availability of pre-mRNA for aberrant splicing	Coupling of transcription kinetics to alternative splicing: high transcription rate promotes inclusion of cryptic exons, while slower Pol II elongation favors canonical splicing [49]
Splicing factor activity	Stress-dependent phosphorylation of SR proteins	Altered exon inclusion/exclusion patterns	Cellular stress alters SR protein phosphorylation and splicing decisions (Biamonti & Caceres) [81]
RNA polymerase II kinetics	Slower elongation under metabolic stress	Reduced generation of cryptic splice sites	Pol II elongation speed affects alternative splicing decisions (Ip & Blencowe) [82]
Nuclear localization	Indirect modulation via nuclear cofactor availability	Possible attenuation of AR-V7 transcriptional output	AR nuclear dynamics and cofactor influence on splice variant function: Dehm & Tindall review on AR variants [58]
Downstream gene programs	Metabolic pathway suppression	Reduced support for CRPC survival	AR-V7 drives metabolic and DNA repair gene networks, linking splicing to metabolic adaptation (Sharp et al.) [59]

**Table 3:** Global Translational Impact of Intermittent Fasting in Prostate Cancer: Directional Biomarker Changes and In Vivo Evidence.

Biomarker	Direction After IF	In Vivo Evidence	Model	Reference
AMPK (p-AMPK)	↑	Fasting activates AMPK in vivo via nutrient deprivation & energy stress	IF/FMD preclinical evidence	[113]
mTOR (p-mTOR, p-S6K)	↓	Reduced mTOR signaling after fasting/FMD	Mouse models	[30]
IGF-1 (serum)	↓	IGF-1 decreased by FMD/fasting	Clinical/murine	[107]
Insulin	↓	Fasting reduces insulin in humans	Clinical	[14]
β-HB	↑	Fasting increases ketone production	Clinical	[14]
AR expression	↓ (via mTOR suppression)	mTOR inhibition linked with decreased AR translation	Preclinical association mechanistic	[114]
PSA	↓	Lower PSA with metabolic modulation contexts	Clinical PCa metabolic studies	[115,116]
GLUT1	↓	Reduced glucose transporter expression under fasting	Preclinical	[117]
HK2	↓	Downregulated under glucose restriction	Preclinical	[117]
CPT1A	↑	FAO upregulated under fasting	Preclinical	[14]
PGC-1α	↑	Mitochondrial adaptation increased with fasting	Preclinical	[14]
8-oxo-dG	↓	Oxidative DNA damage reduced in long-term low nutrient states	Preclinical/aging	[14]
NRF2 targets (NQO1, HMOX1)	↑	Fasting induces NRF2 antioxidative response	Mouse/human metabolic study	[113]
GSH/GSSG ratio	↑	Redox balance improved under fasting/CR-related metabolic states	Animal metabolic studies	[14]
SOD2	↑	Mitochondrial antioxidant defenses increase with IF/CR	Preclinical metabolism	[14]
LC3B-II (autophagy)	↑	Fasting induces autophagy markers in vivo	Preclinical IF models	[117]
Lean body mass (DEXA)	↓/no change	IF significantly decreases body weight and BMI, but does not significantly reduce lean body mass compared with control diets in human trials	Clinical	[118]
Fat mass	↓	Reduced fat mass with IF	Clinical	[107]

Abbreviations: p-mTOR, phosphorylated mTOR; p-S6K, phosphorylated ribosomal protein S6 kinase; β-HB, β-hydroxybutyrate; PSA, prostate-specific antigen; GLUT1, glucose transporter 1; HK2, hexokinase 2; CPT1A, carnitine palmitoyltransferase 1A; 8-oxo-dG, 8-oxo-2′-deoxyguanosine; NQO1, NAD(P)H quinone dehydrogenase 1; HMOX1, heme oxygenase 1; GSH/GSSG, reduced/oxidized glutathione ratio; SOD2, superoxide dismutase 2; LC3B-II, microtubule-associated protein 1 light chain 3 beta, lipidated form; DEXA, dual-energy X-ray absorptiometry. Note: Direct clinical evidence linking each listed biomarker to IF-mediated modulation of AR signaling in PCa remains limited. Where IF-specific data are lacking, references are provided to support the established biological roles of these biomarkers in AR signaling, metabolic regulation, mitochondrial function, redox homeostasis, or treatment response. Collectively, these data provide a mechanistic rationale for their inclusion as candidate biomarkers in future IF-based translational and clinical studies.

**Table 4:** Biomarkers of androgen receptor (AR) signaling and activity in the context of intermittent fasting [16,55,56,119,120].

Biomarker	Sample Type	Rationale	Disease Stage Applicability	IF Regimen Type Relevance
Total AR protein	Tumor biopsy (IHC/WB)	Reduced AR abundance reflects impaired translation and stability under metabolic stress	Localized, mHSPC, mCRPC	ADF, FMD
Nuclear AR localization	Tumor biopsy (IHC/IF)	Decreased nuclear AR indicates reduced transcriptional activity	Localized, mHSPC, mCRPC	ADF, FMD
AR target genes (*PSA*, *TMPRSS2*, *FKBP5*)	Tumor tissue, qPCR	Functional readout of AR transcriptional output	Localized, mHSPC, mCRPC	TRE, ADF, FMD
Serum PSA dynamics	Blood	Clinically established marker of AR-driven tumor activity	Localized, mHSPC, mCRPC	All IF types
AR-V7 (CTCs)	Liquid biopsy	Marker of ligand-independent AR signaling and therapy resistance	mCRPC	ADF, FMD

Abbreviations: *TMPRSS2*, transmembrane protease serine 2; *FKBP5*, FK506 binding protein 5; IHC, immunohistochemistry; WB, western blot; qPCR, quantitative polymerase chain reaction. Note: Direct clinical evidence linking each listed biomarker to IF-mediated modulation of AR signaling in PCa remains limited. Where IF-specific data are lacking, references are provided to support the established biological roles of these biomarkers in AR signaling, metabolic regulation, mitochondrial function, redox homeostasis, or treatment response. Collectively, these data provide a mechanistic rationale for their inclusion as candidate biomarkers in future IF-based translational and clinical studies.

**Table 5:** Metabolic and nutrient-sensing biomarkers reflecting systemic and intratumoral responses to intermittent fasting [84,121,122,123].

Biomarker	Sample Type	Rationale	Disease Stage Applicability	IF Regimen Type Relevance
P-AMPK (Thr172)	Tumor biopsy (IHC/WB)	Indicator of cellular energy stress and fasting response	Localized, mHSPC, mCRPC	TRE, ADF, FMD
P-mTOR/p-S6	Tumor biopsy (IHC)	Suppression reflects inhibition of anabolic growth signaling	Localized, mHSPC, mCRPC	ADF, FMD
Intratumoral amino acid levels	Tumor tissue (metabolomics)	Predict translational capacity of AR and other oncogenic proteins	mHSPC, mCRPC	ADF, FMD
Circulating glucose, insulin, IGF-1	Blood	Systemic metabolic adaptation to IF	Localized, mHSPC, mCRPC	All IF types
Lipogenic enzymes (FASN, ACC)	Tumor biopsy (IHC/qPCR)	AR-driven metabolic output sensitive to nutrient availability	mHSPC, mCRPC	ADF, FMD

Abbreviations: p-AMPK, phosphorylated 5′-adenosine monophosphate-activated protein kinase; p-S6, phosphorylated ribosomal protein S6; ACC, acetyl-CoA carboxylase. Note: Direct clinical evidence linking each listed biomarker to IF-mediated modulation of AR signaling in PCa remains limited. Where IF-specific data are lacking, references are provided to support the established biological roles of these biomarkers in AR signaling, metabolic regulation, mitochondrial function, redox homeostasis, or treatment response. Collectively, these data provide a mechanistic rationale for their inclusion as candidate biomarkers in future IF-based translational and clinical studies.

**Table 6:** Mitochondrial and bioenergetic biomarkers of metabolic adaptation to intermittent fasting [122,124,125,126,127].

Biomarker	Sample Type	Rationale	Disease Stage Applicability	IF Regimen Type Relevance
OXPHOS complex proteins (CI–CV)	Tumor biopsy (IHC)	Shift toward mitochondrial respiration	mHSPC, mCRPC	ADF, FMD
PGC-1α	Tumor biopsy (qPCR/IHC)	Marker of mitochondrial biogenesis	mHSPC, mCRPC	TRE, ADF, FMD
CPT1A	Tumor biopsy (qPCR/IHC)	Fatty acid oxidation and metabolic rewiring	mHSPC, mCRPC	ADF, FMD
Mitophagy markers (PINK1, Parkin)	Tumor biopsy (WB/Immunofluorescence)	Mitochondrial quality control under stress	mHSPC, mCRPC	ADF, FMD

Abbreviations: OXPHOS, oxidative phosphorylation; CI–CV, complexes I–V of the electron transport chain. Note: Direct clinical evidence linking each listed biomarker to IF-mediated modulation of AR signaling in PCa remains limited. Where IF-specific data are lacking, references are provided to support the established biological roles of these biomarkers in AR signaling, metabolic regulation, mitochondrial function, redox homeostasis, or treatment response. Collectively, these data provide a mechanistic rationale for their inclusion as candidate biomarkers in future IF-based translational and clinical studies.

**Table 7:** Biomarkers of redox homeostasis and oxidative stress under intermittent fasting conditions [128,129,130,131].

Biomarker	Sample Type	Rationale	Disease Stage Applicability	IF Regimen Type Relevance
8-oxo-dG	Tumor biopsy (IHC)	Oxidative DNA damage	mHSPC, mCRPC	ADF, FMD, CR
NRF2 target genes (*NQO1*, *HMOX1*)	Tumor tissue (qPCR)	Activation of antioxidant response	Localized, mHSPC, mCRPC	TRE, ADF, FMD
GSH/GSSG ratio	Tumor tissue or plasma (metabolomics)	Cellular redox buffering capacity	Localized, mHSPC, mCRPC	TRE, ADF, FMD, CR
SOD2, GPX4	Tumor biopsy (IHC)	Mitochondrial antioxidant defense	mHSPC, mCRPC	ADF, FMD

Note: Direct clinical evidence linking each listed biomarker to IF-mediated modulation of AR signaling in PCa remains limited. Where IF-specific data are lacking, references are provided to support the established biological roles of these biomarkers in AR signaling, metabolic regulation, mitochondrial function, redox homeostasis, or treatment response. Collectively, these data provide a mechanistic rationale for their inclusion as candidate biomarkers in future IF-based translational and clinical studies.

**Table 8:** Biomarkers of therapeutic response and resistance associated with intermittent fasting [132,133,134,135].

Biomarker	Sample Type	Rationale	Disease Stage Applicability	IF Regimen Type Relevance
Ki-67	Tumor biopsy (IHC)	Proliferative index	Localized, mHSPC, mCRPC	TRE, ADF, FMD
Cleaved caspase-3	Tumor biopsy (IHC)	Apoptosis induction	Localized, mHSPC, mCRPC	TRE, ADF, FMD
p-γH2AX	Tumor biopsy (IHC)	DNA damage and replication stress	mHSPC, mCRPC	ADF, FMD
ctDNA	Liquid biopsy	Tumor burden and clonal evolution	mHSPC, mCRPC	All IF types

Abbreviations: Ki-67, marker of cellular proliferation; p-γH2AX, phosphorylated histone H2AX, marker of DNA double-strand breaks; ctDNA, circulating tumor DNA. Note: Direct clinical evidence linking each listed biomarker to IF-mediated modulation of AR signaling in PCa remains limited. Where IF-specific data are lacking, references are provided to support the established biological roles of these biomarkers in AR signaling, metabolic regulation, mitochondrial function, redox homeostasis, or treatment response. Collectively, these data provide a mechanistic rationale for their inclusion as candidate biomarkers in future IF-based translational and clinical studies.

## Data Availability

No new data were created or analyzed in this study. Data sharing is not applicable to this article.

## Abbreviations

The following abbreviations are used in this manuscript:

8-oxo-dG	8-oxo-2′-deoxyguanosine
ACACA	acetyl-CoA carboxylase alpha
ACC	acetyl-CoA carboxylase
ADF	alternate day fasting
ADT	androgen deprivation therapy
AKT	v-akt murine thymoma viral oncogene homolog
AMPK	AMP-activated protein kinase
AR	androgen receptor
ARE	androgen response elements
ASF/SF2	alternative splicing factor/splicing factor 2
ATP	adenosine triphosphate
BIA	bioelectrical impedance analysis
β-HB	β-hydroxybutyrate
CI–CV	complexes I–V of the electron transport chain
CLK	CDC-like kinase
CPT1A	carnitine palmitoyltransferase 1A
CR	chronic caloric restriction
CRPC	castration-resistant prostate cancer
CTCs	circulating tumor cells
ctDNA	circulating tumor DNA
DEXA	dual-energy X-ray absorptiometry
eIF2α	eukaryotic translation initiation factor 2 subunit alpha
FAO	fatty acid oxidation
FASN	fatty acid synthase
FKBP5	FK506 binding protein 5
FMD	fasting mimicking diets
GCN2	general control nonderepressible 2
GPX4	glutathione peroxidase 4
GSH/GSSG	reduced/oxidized glutathione ratio
HMOX1	heme oxygenase 1
hnRNP	heterogeneous nuclear ribonucleoprotein
HSP90	heat shock protein 90
IF	intermittent fasting
IGF-1	insulin-like growth factor 1
IHC	immunohistochemistry
KAT5	lysine acetyltransferase 5
Ki-67	marker of cellular proliferation
LBD	ligand-binding domain
LC3B-II	microtubule-associated protein 1 light chain 3 beta, lipidated form
mCRPC	metastatic castration-resistant prostate cancer
mHSPC	metastatic hormone-sensitive prostate cancer
NADPH	nicotinamide adenine dinucleotide phosphate
NQO1	NAD(P)H quinone dehydrogenase 1
NRF2	nuclear factor erythroid 2-related factor 2
OXPHOS	oxidative phosphorylation
p38 MAPK	p38 mitogen-activated protein kinase
p-AMPK	phosphorylated AMP-activated protein kinase
PCa	prostate cancer
PGC-1α	peroxisome proliferator-activated receptor gamma coactivator-1 alpha
PI3K	phosphoinositide 3-kinase
PINK1	PTEN-induced kinase 1
p-mTOR	phosphorylated mechanistic target of rapamycin
p-S6	phosphorylated ribosomal protein S6
p-S6K	phosphorylated ribosomal protein S6 kinase
PTM	post-translational modifications
p-γH2AX	phosphorylated histone H2AX, marker of DNA double-strand breaks
RNA	ribonucleic acid
ROS	reactive oxygen species
SCD1	stearoyl-CoA desaturase 1
SIRT1	sirtuin 1
SOD2	superoxide dismutase 2
SREBF1	sterol regulatory element binding transcription factor 1
SREBP-1	sterol regulatory element binding protein-1
SRSF1	serine and arginine rich splicing factor 1
TMPRSS2	transmembrane protease serine 2
TRE	time-restricted eating
U2AF65	U2 snRNP auxiliary factor 65 kDa subunit
WB	Western blot

## References

[1] Bray F., Laversanne M., Sung H., Ferlay J., Siegel R.L., Soerjomataram I., Jemal A. (2024). Global Cancer Statistics 2022: GLOBOCAN Estimates of Incidence and Mortality Worldwide for 36 Cancers in 185 Countries. CA Cancer J. Clin..

[2] Cancer Stat Facts: Prostate Cancer. https://seer.cancer.gov/statfacts/html/prost.html.

[3] Tennakoon J.B., Shi Y., Han J.J., Tsouko E., White M.A., Burns A.R., Zhang A., Xia X., Ilkayeva O.R., Xin L. (2014). Androgens Regulate Prostate Cancer Cell Growth via an AMPK-PGC-1α-Mediated Metabolic Switch. Oncogene.

[4] Adzavon Y.M., Culig Z., Sun Z. (2025). Interactions between Androgen and IGF1 Axes in Prostate Tumorigenesis. Nat. Rev. Urol..

[5] Li C., Cheng D., Li P. (2025). Androgen Receptor Dynamics in Prostate Cancer: From Disease Progression to Treatment Resistance. Front. Oncol..

[6] Chukhu M., Dahiya U.R., Heemers H.V. (2025). Evolving Roles for the Androgen Receptor and Its Protein Interactome in Castration-Resistant Prostate Cancer. Oncogene.

[7] Chetta P., Zadra G. (2021). Metabolic Reprogramming as an Emerging Mechanism of Resistance to Endocrine Therapies in Prostate Cancer. Cancer Drug Resist..

[8] Bernard-Tessier A., Naoun N., Barraud S., Flippot R., Pobel C., Rey M., Baldini C., Massard C., Patrikidou A., Fuerea A. (2025). The Future of Androgen Receptor Targeting in Prostate Cancer: Third-Generation Inhibitors and Beyond. Ther. Adv. Med. Oncol..

[9] Huang F., Li K., Shevach J.W., Wang Q. (2026). Emerging Therapies to Overcome Antiandrogen Resistance and beyond in Lethal Prostate Cancer. J. Natl. Cancer Cent..

[10] Pujana-Vaquerizo M., Bozal-Basterra L., Carracedo A. (2024). Metabolic Adaptations in Prostate Cancer. Br. J. Cancer.

[11] Gonthier K., Poluri R.T.K., Audet-Walsh É. (2019). Functional Genomic Studies Reveal the Androgen Receptor as a Master Regulator of Cellular Energy Metabolism in Prostate Cancer. J. Steroid Biochem. Mol. Biol..

[12] Uo T., Sprenger C.C., Plymate S.R. (2020). Androgen Receptor Signaling and Metabolic and Cellular Plasticity During Progression to Castration Resistant Prostate Cancer. Front. Oncol..

[13] Wanjari U.R., Mukherjee A.G., Gopalakrishnan A.V., Murali R., Dey A., Vellingiri B., Ganesan R. (2023). Role of Metabolism and Metabolic Pathways in Prostate Cancer. Metabolites.

[14] Xie Y., Ye H., Liu Z., Liang Z., Zhu J., Zhang R., Li Y. (2024). Fasting as an Adjuvant Therapy for Cancer: Mechanism of Action and Clinical Practice. Biomolecules.

[15] Fanti M., Longo V.D. (2024). Nutrition, GH/IGF-1 Signaling, and Cancer. Endocr.-Relat. Cancer.

[16] Blaževitš O., Di Tano M., Longo V.D. (2023). Fasting and Fasting Mimicking Diets in Cancer Prevention and Therapy. Trends Cancer.

[17] Caprara G., Pallavi R., Sanyal S., Pelicci P.G. (2025). Dietary Restrictions and Cancer Prevention: State of the Art. Nutrients.

[18] Cordova R.A., Elbanna M., Rupert C., Orsi S.A., Sommers N.R., Klunk A.J., Shen L., Misra J., Hanquier J.N., Tsompana M. (2025). Caloric Restriction Enhances the Efficacy of Anti-Androgen Therapy in Prostate Cancer by Inhibiting Androgen Receptor Translation. Cancer Res..

[19] Shafi A.A., Putluri V., Arnold J.M., Tsouko E., Maity S., Roberts J.M., Coarfa C., Frigo D.E., Putluri N., Sreekumar A. (2015). Differential Regulation of Metabolic Pathways by Androgen Receptor (AR) and Its Constitutively Active Splice Variant, AR-V7, in Prostate Cancer Cells. Oncotarget.

[20] Yin L., Qi S., Zhu Z. (2023). Advances in Mitochondria-Centered Mechanism behind the Roles of Androgens and Androgen Receptor in the Regulation of Glucose and Lipid Metabolism. Front. Endocrinol..

[21] Mah C.Y., Nassar Z.D., Swinnen J.V., Butler L.M. (2020). Lipogenic Effects of Androgen Signaling in Normal and Malignant Prostate. Asian J. Urol..

[22] Zadra G., Ribeiro C.F., Chetta P., Ho Y., Cacciatore S., Gao X., Syamala S., Bango C., Photopoulos C., Huang Y. (2019). Inhibition of de Novo Lipogenesis Targets Androgen Receptor Signaling in Castration-Resistant Prostate Cancer. Proc. Natl. Acad. Sci. USA.

[23] Butler L., Perone Y., Dehairs J., Lupien L.E., de Laat V., Talebi A., Loda M., Kinlaw W.B., Swinnen J.V. (2020). Lipids and Cancer: Emerging Roles in Pathogenesis, Diagnosis and Therapeutic Intervention. Adv. Drug Deliv. Rev..

[24] Tan K.N., Avery V.M., Carrasco-Pozo C. (2020). Metabolic Roles of Androgen Receptor and Tip60 in Androgen-Dependent Prostate Cancer. Int. J. Mol. Sci..

[25] Du H., Xu T., Yu S., Wu S., Zhang J. (2025). Mitochondrial Metabolism and Cancer Therapeutic Innovation. Signal Transduct. Target. Ther..

[26] Yasir S.J., Saif D. The Role of Mitochondrial Stress in Cancer Progression and Therapeutic Resistance. https://www.researchgate.net/publication/391157104_The_Role_of_Mitochondrial_Stress_in_Cancer_Progression_and_Therapeutic_Resistance.

[27] Gillis J.L., Hinneh J.A., Ryan N.K., Irani S., Moldovan M., Quek L.-E., Shrestha R.K., Hanson A.R., Xie J., Hoy A.J. (2021). A Feedback Loop between the Androgen Receptor and 6-Phosphogluoconate Dehydrogenase (6PGD) Drives Prostate Cancer Growth. eLife.

[28] Lu J.P., Monardo L., Bryskin I., Hou Z.F., Trachtenberg J., Wilson B.C., Pinthus J.H. (2010). Androgens Induce Oxidative Stress and Radiation Resistance in Prostate Cancer Cells Though NADPH Oxidase. Prostate Cancer Prostatic Dis..

[29] Sutton E.F., Beyl R., Early K.S., Cefalu W.T., Ravussin E., Peterson C.M. (2018). Early Time-Restricted Feeding Improves Insulin Sensitivity, Blood Pressure, and Oxidative Stress Even without Weight Loss in Men with Prediabetes. Cell Metab..

[30] Brandhorst S., Choi I.Y., Wei M., Cheng C.W., Sedrakyan S., Navarrete G., Dubeau L., Yap L.P., Park R., Vinciguerra M. (2015). A Periodic Diet That Mimics Fasting Promotes Multi-System Regeneration, Enhanced Cognitive Performance, and Healthspan. Cell Metab..

[31] Di Biase S., Lee C., Brandhorst S., Manes B., Buono R., Cheng C.-W., Cacciottolo M., Martin-Montalvo A., de Cabo R., Wei M. (2016). Fasting-Mimicking Diet Reduces HO-1 to Promote T Cell-Mediated Tumor Cytotoxicity. Cancer Cell.

[32] Longo V.D., Fontana L. (2010). Calorie Restriction and Cancer Prevention: Metabolic and Molecular Mechanisms. Trends Pharmacol. Sci..

[33] Nogueira L.M., Lavigne J.A., Chandramouli G.V.R., Lui H., Barrett J.C., Hursting S.D. (2012). Dose-Dependent Effects of Calorie Restriction on Gene Expression, Metabolism, and Tumor Progression Are Partially Mediated by Insulin-like Growth Factor-1. Cancer Med..

[34] Bendykowska M., Gromadzka G., Bendykowska M., Gromadzka G. (2025). Monitoring the Biological Impact and Therapeutic Potential of Intermittent Fasting in Oncology: Assessing Strategies and Clinical Translational Challenges. Diagnostics.

[35] Faris M.E., Alkawamleh D.H., Madkour M.I. (2026). Unraveling the Impact of Intermittent Fasting in Cancer Prevention, Mitigation, and Treatment: A Narrative Review. J. Nutr. Oncol..

[36] Vernieri C., Ligorio F., Tripathy D., Longo V.D. (2024). Cyclic Fasting-Mimicking Diet in Cancer Treatment: Preclinical and Clinical Evidence. Cell Metab..

[37] Bowers L.W., Rossi E.L., O’Flanagan C.H., deGraffenried L.A., Hursting S.D. (2015). The Role of the Insulin/IGF System in Cancer: Lessons Learned from Clinical Trials and the Energy Balance-Cancer Link. Front. Endocrinol..

[38] Wilker E., Lu J., Rho O., Carbajal S., Beltrán L., DiGiovanni J. (2005). Role of PI3K/Akt Signaling in Insulin-like Growth Factor-1 (IGF-1) Skin Tumor Promotion. Mol. Carcinog..

[39] Davaadelger B., Duan L., Perez R.E., Gitelis S., Maki C.G. (2016). Crosstalk between the IGF-1R/AKT/mTORC1 Pathway and the Tumor Suppressors P53 and P27 Determines Cisplatin Sensitivity and Limits the Effectiveness of an IGF-1R Pathway Inhibitor. Oncotarget.

[40] Werner H. (2023). The IGF1 Signaling Pathway: From Basic Concepts to Therapeutic Opportunities. Int. J. Mol. Sci..

[41] Lee D., Martinez B., Crocker D.E., Ortiz R.M. (2017). Fasting Increases the Phosphorylation of AMPK and Expression of Sirtuin1 in Muscle of Adult Male Northern Elephant Seals (*Mirounga angustirostris*). Physiol. Rep..

[42] Goldstein I., Hager G.L. (2015). Transcriptional and Chromatin Regulation during Fasting—The Genomic Era. Trends Endocrinol. Metab..

[43] Fan Y., Peng X., Ebrahimi M., Tabassum N.I., Cheng X., Kumar Y., Liu Y.U., Chen G., Okun E., Johns T.G. (2025). Intermittent Fasting Reprograms Chromatin Accessibility to Modulate Gene Expression in Brain and Muscle. bioRxiv.

[44] Stelloo S., Bergman A., Zwart W. (2019). Androgen Receptor Enhancer Usage and the Chromatin Regulatory Landscape in Human Prostate Cancers. Endocr.-Relat. Cancer.

[45] Fu M., Rao M., Wang C., Sakamaki T., Wang J., Di Vizio D., Zhang X., Albanese C., Balk S., Chang C. (2003). Acetylation of Androgen Receptor Enhances Coactivator Binding and Promotes Prostate Cancer Cell Growth. Mol. Cell. Biol..

[46] Gioeli D., Paschal B.M. (2012). Post-Translational Modification of the Androgen Receptor. Mol. Cell. Endocrinol..

[47] Brandhorst S., Longo V.D. (2016). Fasting and Caloric Restriction in Cancer Prevention and Treatment. Metabolism in Cancer; Recent Results in Cancer Research.

[48] Fan J., Xu Y. (2026). Molecular Mechanisms Underlying the Lifespan and Healthspan Benefits of Dietary Restriction across Species. Front. Genet..

[49] Longo V.D., Mattson M.P. (2014). Fasting: Molecular Mechanisms and Clinical Applications. Cell Metab..

[50] Jurmeister S., Ramos-Montoya A., Neal D.E., Fryer L.G.D. (2014). Transcriptomic Analysis Reveals Inhibition of Androgen Receptor Activity by AMPK in Prostate Cancer Cells. Oncotarget.

[51] Audet-Walsh É., Dufour C.R., Yee T., Zouanat F.Z., Yan M., Kalloghlian G., Vernier M., Caron M., Bourque G., Scarlata E. (2017). Nuclear mTOR Acts as a Transcriptional Integrator of the Androgen Signaling Pathway in Prostate Cancer. Genes Dev..

[52] Giguere V. (2020). DNA-PK, Nuclear mTOR, and the Androgen Pathway in Prostate Cancer. Trends Cancer.

[53] Shen M., Zhang Z., Ratnam M., Dou Q.P. (2014). The Interplay of AMP-Activated Protein Kinase and Androgen Receptor in Prostate Cancer Cells. J. Cell. Physiol..

[54] Anzules J.M., Sayegh M., Li Y.R. (2025). Exploiting AR-Synergistic Metabolic Vulnerabilities in Prostate Cancer. Cancer Res..

[55] Hu R., Dunn T.A., Wei S., Isharwal S., Veltri R.W., Humphreys E., Han M., Partin A.W., Vessella R.L., Isaacs W.B. (2009). Ligand-Independent Androgen Receptor Variants Derived from Splicing of Cryptic Exons Signify Hormone-Refractory Prostate Cancer. Cancer Res..

[56] Antonarakis E.S., Lu C., Wang H., Luber B., Nakazawa M., Roeser J.C., Chen Y., Mohammad T.A., Chen Y., Fedor H.L. (2014). AR-V7 and Resistance to Enzalutamide and Abiraterone in Prostate Cancer. N. Engl. J. Med..

[57] Chan S., Li Y., Dehm S. (2012). Androgen Receptor Splice Variants Activate Androgen Receptor Target Genes and Support Aberrant Prostate Cancer Cell Growth Independent of Canonical Androgen Receptor Nuclear Localization Signal. J. Biol. Chem..

[58] Dehm S.M., Tindall D.J. (2011). Alternatively Spliced Androgen Receptor Variants. Endocr. Relat. Cancer.

[59] Sharp A., Coleman I., Yuan W., Sprenger C., Dolling D., Rodrigues D.N., Russo J.W., Figueiredo I., Bertan C., Seed G. (2019). Androgen Receptor Splice Variant-7 Expression Emerges with Castration Resistance in Prostate Cancer. J. Clin. Investig..

[60] Donega S., Gorospe M., Ferrucci L. (2026). Exploring Splicing-Energy Axis Associations to Diet and Longevity. Aging Cell.

[61] Heinlein C.A., Chang C. (2004). Androgen Receptor in Prostate Cancer. Endocr. Rev..

[62] Zambuzzi W.F., Ferreira M.R., Wang Z., Peppelenbosch M.P. (2025). A Biochemical View on Intermittent Fasting’s Effects on Human Physiology—Not Always a Beneficial Strategy. Biology.

[63] Vasim I., Majeed C.N., DeBoer M.D. (2022). Intermittent Fasting and Metabolic Health. Nutrients.

[64] Zhu X., Wang X., Wang J., Du L., Zhang Z.-N., Zhou D., Han J., Luan B. (2024). Intermittent Fasting-Induced Orm2 Promotes Adipose Browning via the GP130/IL23R-P38 Cascade. Adv. Sci..

[65] Elechi J.O.G., de Mendonça C.R., de Araújo Bandeira V.C., Da Silva M.S.P., de Melo A.P.R., Guedes R.C.A., Hirabara S.M., Cione E., de Vasconcelos D.A.A. (2026). Exploring Recent Insights on Intermittent Fasting in Regulating Glucocorticoid Levels and Diet-Induced Metabolic Disorders with Focus on MAFLD and Hepatic Outcomes. Mol. Cell. Endocrinol..

[66] Gioeli D., Black B.E., Gordon V., Spencer A., Kesler C.T., Eblen S.T., Paschal B.M., Weber M.J. (2006). Stress Kinase Signaling Regulates Androgen Receptor Phosphorylation, Transcription, and Localization. Mol. Endocrinol..

[67] Bogoyevitch M.A. (2000). Signalling via Stress-Activated Mitogen-Activated Protein Kinases in the Cardiovascular System. Cardiovasc. Res..

[68] Plotnikov A., Zehorai E., Procaccia S., Seger R. (2011). The MAPK Cascades: Signaling Components, Nuclear Roles and Mechanisms of Nuclear Translocation. Biochim. Biophys. Acta (BBA) Mol. Cell Res..

[69] Ishii T., Warabi E., Mann G.E. (2023). Stress Activated MAP Kinases and Cyclin-Dependent Kinase 5 Mediate Nuclear Translocation of Nrf2 via Hsp90α-Pin1-Dynein Motor Transport Machinery. Antioxidants.

[70] der Steen T.V., Tindall D.J., Huang H. (2013). Posttranslational Modification of the Androgen Receptor in Prostate Cancer. Int. J. Mol. Sci..

[71] Shah K., Bradbury N.A. (2015). Kinase Modulation of Androgen Receptor Signaling: Implications for Prostate Cancer. Cancer Cell Microenviron..

[72] Cordova R.A., Misra J., Amin P.H., Klunk A.J., Damayanti N.P., Carlson K.R., Elmendorf A.J., Kim H.-G., Mirek E.T., Elzey B.D. (2022). GCN2 eIF2 Kinase Promotes Prostate Cancer by Maintaining Amino Acid Homeostasis. Elife.

[73] Saporita A.J., Ai J., Wang Z. (2007). The Hsp90 Inhibitor, 17-AAG, Prevents the Ligand-Independent Nuclear Localization of Androgen Receptor in Refractory Prostate Cancer Cells. Prostate.

[74] Tien A.H., Sadar M.D. (2019). Keys to Unlock Androgen Receptor Translocation. J. Biol. Chem..

[75] Ni L., Llewellyn R., Kesler C.T., Kelley J.B., Spencer A., Snow C.J., Shank L., Paschal B.M. (2013). Androgen Induces a Switch from Cytoplasmic Retention to Nuclear Import of the Androgen Receptor. Mol. Cell. Biol..

[76] Tan M.E., Li J., Xu H.E., Melcher K., Yong E. (2015). Androgen Receptor: Structure, Role in Prostate Cancer and Drug Discovery. Acta Pharmacol. Sin..

[77] Cai C., Yuan X., Balk S.P. (2013). Androgen Receptor Epigenetics. Transl. Androl. Urol..

[78] Tietz K.T., Dehm S.M. (2020). Androgen Receptor Variants: RNA-Based Mechanisms and Therapeutic Targets. Hum. Mol. Genet..

[79] Liu N., van der Ende F., van de Water B., Le Dévédec S.E. (2026). Decoding SR Protein Regulation: Kinases, Phosphatases, and Therapeutic Targeting Strategies. Cell. Oncol..

[80] Watson P.A., Arora V.K., Sawyers C.L. (2015). Emerging Mechanisms of Resistance to Androgen Receptor Inhibitors in Prostate Cancer. Nat. Rev. Cancer.

[81] Biamonti G., Caceres J.F. (2009). Cellular Stress and RNA Splicing. Trends Biochem. Sci..

[82] Ip J.Y., Schmidt D., Pan Q., Ramani A.K., Fraser A.G., Odom D.T., Blencowe B.J. (2011). Global Impact of RNA Polymerase II Elongation Inhibition on Alternative Splicing Regulation. Genome Res..

[83] Massie C.E., Lynch A., Ramos-Montoya A., Boren J., Stark R., Fazli L., Warren A., Scott H., Madhu B., Sharma N. (2011). The Androgen Receptor Fuels Prostate Cancer by Regulating Central Metabolism and Biosynthesis. EMBO J..

[84] Hardie D.G. (2014). AMP-Activated Protein Kinase: Maintaining Energy Homeostasis at the Cellular and Whole-Body Levels. Annu. Rev. Nutr..

[85] González A., Hall M.N., Lin S.-C., Hardie D.G. (2020). AMPK and TOR: The Yin and Yang of Cellular Nutrient Sensing and Growth Control. Cell Metab..

[86] Zhang W., Zhu J., Efferson C.L., Ware C., Tammam J., Angagaw M., Laskey J., Bettano K.A., Kasibhatla S., Reilly J.F. (2009). Inhibition of Tumor Growth Progression by Antiandrogens and mTOR Inhibitor in a Pten-Deficient Mouse Model of Prostate Cancer. Cancer Res..

[87] Corbin J.M., Ruiz-Echevarría M.J. (2016). One-Carbon Metabolism in Prostate Cancer: The Role of Androgen Signaling. Int. J. Mol. Sci..

[88] Wang Q., Bailey C.G., Ng C., Tiffen J., Thoeng A., Minhas V., Lehman M.L., Hendy S.C., Buchanan G., Nelson C.C. (2011). Androgen Receptor and Nutrient Signaling Pathways Coordinate the Demand for Increased Amino Acid Transport during Prostate Cancer Progression. Cancer Res..

[89] Wek R.C. (2018). Role of eIF2α Kinases in Translational Control and Adaptation to Cellular Stress. Cold Spring Harb. Perspect. Biol..

[90] Misra J., Carlson K.R., Spandau D.F., Wek R.C. (2024). Multiple Mechanisms Activate GCN2 eIF2 Kinase in Response to Diverse Stress Conditions. Nucleic Acids Res..

[91] Falcón P., Brito Á., Escandón M., Roa J.F., Martínez N.W., Tapia-Godoy A., Farfán P., Matus S. (2025). GCN2-Mediated eIF2α Phosphorylation Is Required for Central Nervous System Remyelination. Int. J. Mol. Sci..

[92] Pakos-Zebrucka K., Koryga I., Mnich K., Ljujic M., Samali A., Gorman A.M. (2016). The Integrated Stress Response. EMBO Rep..

[93] Mazor K.M., Stipanuk M.H. (2016). GCN2- and eIF2α-Phosphorylation-Independent, but ATF4-Dependent, Induction of CARE-Containing Genes in Methionine-Deficient Cells. Amino Acids.

[94] Nencioni A., Caffa I., Cortellino S., Longo V.D. (2018). Fasting and Cancer: Molecular Mechanisms and Clinical Application. Nat. Rev. Cancer.

[95] Shorning B.Y., Dass M.S., Smalley M.J., Pearson H.B. (2020). The PI3K-AKT-mTOR Pathway and Prostate Cancer: At the Crossroads of AR, MAPK, and WNT Signaling. Int. J. Mol. Sci..

[96] Qi W., Morales C., Cooke L.S., Johnson B., Somer B., Mahadevan D. (2015). Reciprocal Feedback Inhibition of the Androgen Receptor and PI3K as a Novel Therapy for Castrate-Sensitive and -Resistant Prostate Cancer. Oncotarget.

[97] Zhao J.L., Antonarakis E.S., Cheng H.H., George D.J., Aggarwal R., Riedel E., Sumiyoshi T., Schonhoft J.D., Anderson A., Mao N. (2024). Phase 1b Study of Enzalutamide plus CC-115, a Dual mTORC1/2 and DNA-PK Inhibitor, in Men with Metastatic Castration-Resistant Prostate Cancer (mCRPC). Br. J. Cancer.

[98] Schöpf B., Weissensteiner H., Schäfer G., Fazzini F., Charoentong P., Naschberger A., Rupp B., Fendt L., Bukur V., Giese I. (2020). OXPHOS Remodeling in High-Grade Prostate Cancer Involves mtDNA Mutations and Increased Succinate Oxidation. Nat. Commun..

[99] Butler L.M., Centenera M.M., Swinnen J.V. (2016). Androgen Control of Lipid Metabolism in Prostate Cancer: Novel Insights and Future Applications. Endocr. Relat. Cancer.

[100] Caffa I., Spagnolo V., Vernieri C., Valdemarin F., Becherini P., Wei M., Brandhorst S., Zucal C., Driehuis E., Ferrando L. (2020). Fasting-Mimicking Diet and Hormone Therapy Induce Breast Cancer Regression. Nature.

[101] Mondal D., Narwani D., Notta S., Ghaffar D., Mardhekar N., Quadri S.S. (2021). Oxidative Stress and Redox Signaling in CRPC Progression: Therapeutic Potential of Clinically-Tested Nrf2-Activators. Cancer Drug Resist..

[102] Zhao B., Wang J., Chen L., Wang H., Liang C.-Z., Huang J., Xu L.-F. (2023). The Role of Glutamine Metabolism in Castration-Resistant Prostate Cancer. Asian J. Androl..

[103] Erb H.H.H., Polishchuk N., Stasyk O., Kahya U., Weigel M.M., Dubrovska A. (2024). Glutamine Metabolism and Prostate Cancer. Cancers.

[104] Wang Q., Tiffen J., Bailey C.G., Lehman M.L., Ritchie W., Fazli L., Metierre C., Feng Y.J., Li E., Gleave M. (2013). Targeting Amino Acid Transport in Metastatic Castration-Resistant Prostate Cancer: Effects on Cell Cycle, Cell Growth, and Tumor Development. J. Natl. Cancer Inst..

[105] University at Buffalo Intermittent Fasting in Prostate Cancer Patients Receiving Androgen Deprivation Therapy (NCT06172283). ClinicalTrials.gov, Identifier NCT06172283. NCT06172283.

[106] Feldman B.J., Feldman D. (2001). The Development of Androgen-Independent Prostate Cancer. Nat. Rev. Cancer.

[107] Wei M., Brandhorst S., Shelehchi M., Mirzaei H., Cheng C.W., Budniak J., Groshen S., Mack W.J., Guen E., Di Biase S. (2017). Fasting-Mimicking Diet and Markers/Risk Factors for Aging, Diabetes, Cancer, and Cardiovascular Disease. Sci. Transl. Med..

[108] Nguyen P.L., Alibhai S.M.H., Basaria S., D’Amico A.V., Kantoff P.W., Keating N.L., Penson D.F., Rosario D.J., Tombal B., Smith M.R. (2015). Adverse Effects of Androgen Deprivation Therapy and Strategies to Mitigate Them. Eur. Urol..

[109] Sweeney C.J., Chen Y.-H., Carducci M., Liu G., Jarrard D.F., Eisenberger M., Wong Y.-N., Hahn N., Kohli M., Cooney M.M. (2015). Chemohormonal Therapy in Metastatic Hormone-Sensitive Prostate Cancer. N. Engl. J. Med..

[110] Saylor P.J., Smith M.R. (2013). Metabolic Complications of Androgen Deprivation Therapy for Prostate Cancer. J. Urol..

[111] Smith M.R., Finkelstein J.S., McGovern F.J., Zietman A.L., Fallon M.A., Schoenfeld D.A., Kantoff P.W. (2002). Changes in Body Composition during Androgen Deprivation Therapy for Prostate Cancer. J. Clin. Endocrinol. Metab..

[112] Vernieri C., Fuca G., Ligorio F., Huber V., Vingiani A., Iannelli F., Raimondi A., Rinchai D., Frige G., Belfiore A. (2022). Fasting-Mimicking Diet Is Safe and Reshapes Metabolism and Antitumor Immunity in Patients with Cancer. Cancer Discov..

[113] Kulkarni S.R., Donepudi A.C., Xu J., Wei W., Cheng Q.C., Driscoll M.V., Johnson D.A., Johnson J.A., Li X., Slitt A.L. (2014). Fasting Induces Nuclear Factor E2-Related Factor 2 and ATP-Binding Cassette Transporters via Protein Kinase A and Sirtuin-1 in Mouse and Human. Antioxid. Redox Signal..

[114] Zhang J., Deng Y., Khoo B.L. (2020). Fasting to Enhance Cancer Treatment in Models: The next Steps. J. Biomed. Sci..

[115] Mayo Clinic (2022). A Prospective Pilot Study Evaluating the Feasibility of Daily, Long-Term Intermittent Fasting for Men on PSA Surveillance Following Radical Prostatectomy for Localized, High-Risk Prostate Cancer. https://www.patlynk.com/trial/NCT04288336.

[116] Letkiewicz S., Pilis K., Ślęzak A., Pilis A., Pilis W., Żychowska M., Langfort J. (2020). Eight Days of Water-Only Fasting Promotes Favorable Changes in the Functioning of the Urogenital System of Middle-Aged Healthy Men. Nutrients.

[117] Wolska W., Gutowska I., Wszołek A., Żwierełło W. (2025). The Role of Intermittent Fasting in the Activation of Autophagy Processes in the Context of Cancer Diseases. Int. J. Mol. Sci..

[118] Khalafi M., Habibi Maleki A., Symonds M.E., Rosenkranz S.K., Rohani H., Ehsanifar M. (2024). The Effects of Intermittent Fasting on Body Composition and Cardiometabolic Health in Adults with Prediabetes or Type 2 Diabetes: A Systematic Review and Meta-Analysis. Diabetes Obes. Metab..

[119] Van Poppel H., Roobol M.J., Chapple C.R., Catto J.W.F., N’Dow J., Sønksen J., Stenzl A., Wirth M. (2021). Prostate-Specific Antigen Testing as Part of a Risk-Adapted Early Detection Strategy for Prostate Cancer: European Association of Urology Position and Recommendations for 2021. Eur. Urol..

[120] Schröder F.H. (2006). Early Detection of Prostate Cancer. What Do We Tell Our Patients?. Can. J. Urol..

[121] Fay-Watt V., O’Connor S., Roshan D., Romeo A.C., Longo V.D., Sullivan F.J. (2023). The Impact of a Fasting Mimicking Diet on the Metabolic Health of a Prospective Cohort of Patients with Prostate Cancer: A Pilot Implementation Study. Prostate Cancer Prostatic Dis..

[122] Penfold L., Woods A., Pollard A.E., Arizanova J., Pascual-Navarro E., Muckett P.J., Dore M.H., Montoya A., Whilding C., Fets L. (2023). AMPK Activation Protects against Prostate Cancer by Inducing a Catabolic Cellular State. Cell Rep..

[123] Safdie F.M., Dorff T., Quinn D., Fontana L., Wei M., Lee C., Cohen P., Longo V.D. (2009). Fasting and Cancer Treatment in Humans: A Case Series Report. Aging.

[124] Mindikoglu A.L., Abdulsada M.M., Jain A., Jalal P.K., Devaraj S., Wilhelm Z.R., Opekun A.R., Jung S.Y. (2020). Intermittent Fasting from Dawn to Sunset for Four Consecutive Weeks Induces Anticancer Serum Proteome Response and Improves Metabolic Syndrome. Sci. Rep..

[125] Pickrell A.M., Youle R.J. (2015). The Roles of PINK1, Parkin, and Mitochondrial Fidelity in Parkinson’s Disease. Neuron.

[126] Lemasters J.J. (2005). Selective Mitochondrial Autophagy, or Mitophagy, as a Targeted Defense against Oxidative Stress, Mitochondrial Dysfunction, and Aging. Rejuvenation Res..

[127] Villa E., Proïcs E., Rubio-Patiño C., Obba S., Zunino B., Bossowski J.P., Rozier R.M., Chiche J., Mondragón L., Riley J.S. (2017). Parkin-Independent Mitophagy Controls Chemotherapeutic Response in Cancer Cells. Cell Rep..

[128] Tejchman K., Kotfis K., Sieńko J. (2021). Biomarkers and Mechanisms of Oxidative Stress-Last 20 Years of Research with an Emphasis on Kidney Damage and Renal Transplantation. Int. J. Mol. Sci..

[129] Liang X., Weng J., You Z., Wang Y., Wen J., Xia Z., Huang S., Luo P., Cheng Q. (2025). Oxidative Stress in Cancer: From Tumor and Microenvironment Remodeling to Therapeutic Frontiers. Mol. Cancer.

[130] Jomova K., Alomar S.Y., Valko R., Fresser L., Nepovimova E., Kuca K., Valko M. (2026). Interplay of Oxidative Stress and Antioxidant Mechanisms in Cancer Development and Progression. Arch. Toxicol..

[131] Antunes F., Erustes A.G., Costa A.J., Nascimento A.C., Bincoletto C., Ureshino R.P., Pereira G.J.S., Smaili S.S. (2018). Autophagy and Intermittent Fasting: The Connection for Cancer Therapy?. Clinics.

[132] Song Z., Zhou Q., Zhang J.-L., Ouyang J., Zhang Z.-Y. (2024). Marker Ki-67 Is a Potential Biomarker for the Diagnosis and Prognosis of Prostate Cancer Based on Two Cohorts. World J. Clin. Cases.

[133] Brandhorst S. (2021). Fasting and Fasting-Mimicking Diets for Chemotherapy Augmentation. GeroScience.

[134] Li X.Q. (1986). Diagnostic value of the relative width of the action potential-summating potential wave in Menière’s disease. Zhonghua Yi Xue Za Zhi.

[135] Tiwari S., Sapkota N., Han Z. (2022). Effect of Fasting on Cancer: A Narrative Review of Scientific Evidence. Cancer Sci..

[136] International Agency for Research on Cancer (IARC) Global Cancer Observatory. https://gco.iarc.fr/.

[137] Kalam F., James D.L., Li Y.R., Coleman M.F., Kiesel V.A., Cespedes Feliciano E.M., Hursting S.D., Sears D.D., Kleckner A.S. (2023). Intermittent fasting interventions to leverage metabolic and circadian mechanisms for cancer treatment and supportive care outcomes. JNCI Monogr..

[138] Salvadori G., Mirisola M.G., Longo V.D. (2021). Intermittent and Periodic Fasting, Hormones, and Cancer Prevention. Cancers.

[139] Sadria M., Layton A.T. (2021). Interactions among mTORC, AMPK and SIRT: A Computational Model for Cell Energy Balance and Metabolism. Cell Commun. Signal..

[140] Stelmach-Mardas M., Warchoł W., Garczyk A., Warchoł E., Korczak J., Litwiniuk M., Brajer-Luftmann B., Mardas M. (2024). Influence of Androgen Deprivation Therapy on the Development of Sarcopenia in Patients with Prostate Cancer: A Systematic Review. Nutrients.

[141] Nakazawa M., Lu C., Chen Y., Paller C.J., Carducci M.A., Eisenberger M.A., Luo J., Antonarakis E.S. (2015). Serial Blood-Based Analysis of AR-V7 in Men with Advanced Prostate Cancer. Ann. Oncol..

[142] Pereira I.C., Martins J.A., de Sousa D.J.M., de Oliveira Neres M.S., Lima R.S.P., da Silva Sousa J., Monteiro R.L., da Silva F.C.C., Severo J.S., Torres-Leal F.L. (2026). Fasting-Mimicking Diets as a Strategy to Reprogram Tumor Metabolism: A Systematic Review. Eur. J. Nutr..

[143] Study: In Prostate Cancer, Intermittent Fasting Is Found to Enhance Efficacy of Anti-Androgen Therapy. https://www.buffalo.edu/news/releases/2025/08/Prostate-cancer-intermittent-fasting.html.

[144] Piestansky J., Matuskova M., Cizmarova I., Olesova D., Mikus P. (2021). Determination of Branched-Chain Amino Acids in Food Supplements and Human Plasma by a CE-MS/MS Method with Enhanced Resolution. Int. J. Mol. Sci..

[145] Chan J.M., Stampfer M.J., Giovannucci E., Gann P.H., Ma J., Wilkinson P., Hennekens C.H., Pollak M. (1998). Plasma Insulin-Like Growth Factor-I and Prostate Cancer Risk: A Prospective Study. Science.

[146] Yu H., Rohan T. (2000). Role of the Insulin-like Growth Factor Family in Cancer Development and Progression. J. Natl. Cancer Inst..

[147] Pollak M. (2012). The Insulin and Insulin-like Growth Factor Receptor Family in Neoplasia: An Update. Nat. Rev. Cancer.

[148] Shaw R.J., Kosmatka M., Bardeesy N., Hurley R.L., Witters L.A., DePinho R.A., Cantley L.C. (2004). The Tumor Suppressor LKB1 Kinase Directly Activates AMP-Activated Kinase and Regulates Apoptosis in Response to Energy Stress. Proc. Natl. Acad. Sci. USA.

[149] Cantó C., Gerhart-Hines Z., Feige J.N., Lagouge M., Noriega L., Milne J.C., Elliott P.J., Puigserver P., Auwerx J. (2009). AMPK Regulates Energy Expenditure by Modulating NAD+ Metabolism and SIRT1 Activity. Nature.

